# Treatment of established status epilepticus in the elderly - a study protocol for a prospective multicenter double-blind comparative effectiveness trial (ToSEE)

**DOI:** 10.1186/s12883-020-02001-x

**Published:** 2020-12-03

**Authors:** Annekatrin Müller, Anett Schmiedeknecht, Meinhard Mende, Carolin Awissus, Felix Rosenow, Hajo Hamer, Joseph Classen

**Affiliations:** 1 Department of Neurology, University of Leipzig, Leipzig, Germany; 2 Clinical Trial Centre Leipzig, University of Leipzig, Leipzig, Germany; 3 Institute for Medical Informatics, Statistics and Epidemiology, University of Leipzig, Leipzig, Germany; 4 Epilepsy Center Frankfurt Rhein-Main, Department of Neurology, University Hospital Frankfurt and LOEWE Center for Personalized Translational Epilepsy Research (CePTER) Goethe University Frankfurt, Frankfurt, Germany; 5 Department of Neurology, Erlangen Epilepsy Center, University of Erlangen, Friedrich-Alexander University Erlangen-Nürnberg (FAU), Erlangen, Germany

**Keywords:** Benzodiazepine refractory status epilepticus, Elderly, Valproate, Levetiracetam

## Abstract

**Background:**

Status epilepticus (SE) is a common neurological emergency condition that especially affects the elderly and old population. Older people with SE frequently have non-convulsive SE (NCSE) and are also at special risk of suffering a poor outcome. The application of benzodiazepines fails to control SE in about one third of the cases. For benzodiazepine refractory SE (BRSE) in elderly, there is little evidence that would justify the choice of one of the commonly used antiepileptic drugs. The present study aims to generate evidence for the treatment of BRSE in this age group.

**Methods:**

We will conduct a prospective, randomized, double-blind comparative effectiveness study in more than twenty hospitals in Germany over a four-year period. Four hundred and seventy-seven elderly patients (≥ 65 years old) diagnosed with BRSE will be allocated by 1:1 randomization to receive either levetiracetam or valproate. All types of SE will be considered. For the diagnosis NCSE a verification by EEG is required. Levetiracetam or valproate will be administered in one single infusion. The primary endpoint is the stable cessation of ictal activity 15 min after the start of infusion persisting for the following 45 min of observation. EEG recording is maintained over the whole observation period, clinical examinations are conducted in predefined intervals. In case of treatment success patients and study staff remain blinded until 60 min after the start of the infusion. Adverse events will be recorded until the end of the study. EEG data will be reviewed by two external independent experts. To obtain data about the further treatment of SE, intrahospital complications and the functional outcome in the short term the study participants will be observed until the day of discharge or day 30 whichever is earliest.

**Discussion:**

ToSEE is the first study which shall deliver evidence for the SE-therapy in the elderly and old population in a controlled prospective comparator study. By design it also shall collect information about therapy regimes and outcome aspects of this disease.

**Trial registration:**

The trial has been registered at the German Clinical Trials Register on 3 July, 2020 (DRKS00022308, https://www.drks.de/drks_web/navigate.do?navigationId=trial.HTML&TRIAL_ID=DRKS00022308).

## Background

Status epilepticus (SE) is a significant and growing burden in Germany that especially affects the elderly where incidence has its highest peak with 54.5 per 100.000 people over 60 years of age [1]. Older people are also at special risk of suffering a poor outcome, partly because SE may be particularly challenging to treat, due to the nature of the causative brain injury [2]. Beyond the clinically overt types of SE, the nonconvulsive SE (NCSE) is frequent in older people and carries a risk of permanent neurological damage [3]. NCSE is underdiagnosed because of its highly variable clinical presentation and the requirement of electroencephalographical validation.

As longer duration of SE is associated with higher morbidity [4] the treatment maxim “time is brain” applies not only for stroke but also for SE. Although it is the second most frequent neurological emergency, there is a surprising lack of high level evidence regarding treatment strategies after the application of benzodiazepines as first line treatment that fails in approximately 40% or more of the cases [5, 6]. Irrespective of a convulsive or nonconvulsive SE, the commonly used antiepileptic drugs (fos)phenytoin, valproate (VPA), levetiracetam (LEV), phenobarbital and lacosamide are recommended for the treatment of BRSE [7, 8].

In 2019, first data of the multicentre trial “Established Status Epilepticus Treatment Trial-ESETT” were published about the efficacy and tolerability of the three most commonly used drugs LEV, VPA and fosphenytoin (FPHT) in generalized convulsive BRSE.^1^ Each of the three drugs led to seizure cessation and improvement of consciousness in approximately half of the patients with similar incidences of adverse events [9]. As only 50 (13%) patients were older than 65 years and only convulsive SE has been considered in this study, the question about an effective and safe therapy in the elderly remains unanswered. Also, there is no precisely defined pathway for the SE-treatment after the first stage concerning drugs, their dosages or time intervals for application.

To address the lack of evidence, the primary objective of ToSEE is to compare LEV and VPA regarding efficacy and safety in BRSE in the elderly. All types of BRSE will be considered. VPA and LEV deemed best suited to be considered for testing as they combine the best safety profile in elderly patients with documented, albeit insufficient, evidence of their efficacy. In the past, VPA was administered in effective doses from 15 to 45 mg/kg. Safety studies showed a generally low incidence of side effects (< 10%; mainly thrombocytopenia and mild hypotension), that occurred independently of infusion rates. The most severe side-effects are hepatotoxicity and also encephalopathy, which has been observed to rarely follow hepatic dysfunction or hyperammonaemia [10]. LEV displays a low risk of side effects and drug interactions. Side effects included psychiatric disturbances, somnolence, fatigue and headache. In single cases, thrombocytopenia, agitation, delirium and psychosis were described [11, 12]. FPHT is not available in several European countries. Perhaps of even greater significance, FPHT carries the risk of cardiac arrhythmias especially in the elderly with SE who frequently have cardiac disorders. Lacosamide has been ruled out due to the lack of published prospective trials for this condition. Phenobarbital has been ruled out because of its sedative properties and respiratory depression.

ToSEE also shall collect information about this neurological emergency, its complications and outcome aspects in the elderly. Considering the lack of sufficient and robust scientific evidence, the life-threatening nature of the condition and the frequently poor outcome of older patients suffering from BRSE the conduction of this study appears to be both needed and justified.

## Methods/ design

### Study design

“Treatment of Established Status Epilepticus in the Elderly- ToSEE” is a multicenter, prospective, controlled, double-blind, randomized comparative effectiveness phase IV- study with two treatment arms, that will be conducted in emergency and neurological departments in more than 20 hospitals in Germany (EudraCT-No.: 2018–003917-16; DRKS00022308, a list of the trial sites can be found at https://www.drks.de, the World Health Organization Trial Registration Data Set is available at https://www.who.int). We aim to include university hospitals and general hospitals as well to obtain data on a representative part of the German population to improve the generalizability of the results. Subjects will be randomized 1:1 in either the VPA or the LEV treatment arm. Randomization is realized by the trial package and patient identification numbers at each trial site and will be conducted electronically afterwards at the Clinical Trial Centre Leipzig. Over 4 years we plan to enrol 477 patients, and data of 454 patients are planned for analysis. The study protocol was approved by the leading ethics committee (University Leipzig, study protocol version 4.0, 18.05.2020, see Additional File 1) and the federal authority (BfArM). The study is approved to enrol patients who are unable to provide informed consent according to paragraph 41 of the German Medicinal Products Act.

The study is funded by the Federal Ministry of Education and Research (No: 01GL1804). Reporting of the study follows the SPIRIT (Standard Protocol Items: Recommendations for Interventional Trials) 2013 statement and guidelines.

### Study population

Adults at the age of 65 years or older with ongoing convulsive SE (generalized/ focal with impaired consciousness/ focal without impaired consciousness) or NCSE (with coma/ without coma) that do not respond to a treatment with adequate dosages of benzodiazepines are considered for inclusion. Adequate dosages of benzodiazepines in this study are: lorazepam > = 2 mg (intravenous, IV), midazolam > = 5 mg (IV, intranasal, buccal, intramuscular), diazepam 5 mg (IV or rectal) or clonazepam > = 1 mg (IV). Convulsive SE is defined as a seizure that lasts ≥5 min; or 2 or more convulsive seizures without full recovery of neurological baseline status for ≥5 min. NCSE is defined as ongoing EEG patterns consistent with definite or possible NCSE according to the Salzburg criteria [13]. Clinically defined NCSE may be diagnosed under the following circumstances: patients with confusion/ fluctuating mental state and (minimal) rhythmic motor activity (as twitching of the arms, legs, trunk or facial muscles, blinking, tonic eye deviation or nystagmoid eye jerking) or comatose/stuporous state and (minimal) rhythmic motor activity (see above). Clinically defined diagnosis of NCSE is only permitted if EEG is not available or justified for verification of NCSE. In particular, clinical NCSE must not be diagnosed if NSCE is suspected, but EEG is ambiguous. The proportion of patients with NCSE diagnosed without EEG should be limited to 5% of all patients with NCSE. Patients are not eligible if there are known contradictions for the administration of the trial drugs: known or suspected severe liver or pancreatic disease (alcohol addiction, known liver cirrhosis or familial liver diseases, clinical signs of severe liver disease such as ascites or jaundice), coagulopathy (anticoagulants allowed), porphyria, mitochondriopathy and urea cycle disorders, severe kidney disease, and insulin dependent diabetes mellitus. Patients will be excluded if the trial drugs had been administered IV within 24 h before enrolment, if the SE episode has been treated with a second or third line anticonvulsant before enrolment or if there are concomitant therapies that are known to influence the plasma level of VPA, i.e. phenobarbital, phenytoin, and carbapenem antibiotics. In addition, patients will be excluded if their weight is lower than 45 kg, if they suffer from hypoglycaemia, if there is need for acute neurosurgical intervention or if they had undergone cardiopulmonary resuscitation within the last 7 days before enrolment. A former participation in this study or a known participation in another interventional study is criterion for exclusion, too.

### Sample size and power calculation

The meta-analysis of Yasiry and Shorvon (2014) [14] compares cessation of benzodiazepine resistant SE after treatment with one of five substances which included VPA and LEV. In a second meta-analysis, Trinka et al. (2014) [15] examined the efficacy of VPA in therapy of established SE. Third, Alvarez et al. (2011) [16] published results of the registry of benzodiazepine- resistant SE patients. The reported frequencies of cessation of SE (within variable periods) vary widely due to different study designs (randomized clinical trial, prospective open label or retrospective trial, dose schemes, endpoint definition). The cessation rates in the present trial with elderly patients are expected to be lower than those reported by Yasiry and Shorvon (2014): VPA 76% and LEV 68.5%, see e.g., Zelano and Kumlien (2012) [17]. Another article [18] reports 70% response for VPA treatment. New articles [9, 11, 19] do not eliminate the indecision between the standard drugs for stage II. The systematic review and meta-analysis from Sánchez Fernández (2019) [19] report 70% success for VPA and 62% for LEV. In the ESET trial cessation of SE occurred in 47% of the patients assigned to LEV and 46% of the patients assigned to VPA [9]. Omitting the studies of pediatric SE and with first-line treatment of SE, an odds ratio OR = 1.7 in favour of VPA vs. LEV is calculated, which seems much more realistic than the reported OR = 2.7 for SE cessation in Alvarez et al. (2011). The OR = 1.7 applies to cessation frequencies of 60% (VPA) and 47% (LEV). Assuming these rates, a significance level of 5% and a power of 80%, the R (R Core Team 2017) package TrialSize calculates for chi-squared test a sample size of *N* = 454. Assuming a drop-out rate of about 5%, a total of 477 patients are to be randomized.

Issues of feasibility were discussed with collaborating partners via email or phone, with special emphasis on a conservative estimate of the number of eligible patients. Based on the experiences of the participating centres a mean rate of 5 patients per year and centre was estimated. Based on ≥20 participating centres the target goal of recruiting 120 patients per year appears to be feasible.

### Ethical aspects

Considering the present scientific evidence ToSEE offers the so far best available treatments. Due to the nature of the disease and the prior therapy with benzodiazepines, the patients will not be able to provide informed consent before the initiation of the trial therapy. According to the SE subtype the inclusion will be realized in strict adherence to the urgency of treatment. Patients with generalized convulsive SE and convulsive SE with disturbance of consciousness are to be treated immediately so the treating physician decides about inclusion regarding the inclusion and exclusion criteria. Patients suffering from convulsive SE without disturbance of consciousness or nonconvulsive SE fulfil the criteria of an urgent but not immediate treatment indication so a legal or authorized representative if present or an independent medical consultant are to be involved. The first described procedure follows § 41 (1) of the German Medicinal Products Act, both procedures are recommended by the Working Committee of Medical Ethics Committees of the States of the Federal Republic of Germany [20] (https://www.uni-giessen.de/fbz/fb11/dekanat/ethikkommission/nichteinwfpers). Patients initially unable to provide consent must be informed about the clinical study as soon as they are able to do so and will then be asked to provide their written informed consent. If the patient has not regained consciousness 72 h after inclusion into the study, the establishment of a legal care relationship should be initiated at the responsible local court.

### Randomization

Randomization in a 1:1 ratio is performed as block randomization with randomly varying block length stratified by centre. Randomization lists are created by statistical software. Blocks of randomly chosen length are created as realizations of random samples and stringed together to a list. The Clinical Trial Centre Leipzig delivers one randomization list per centre each to the central pharmacy Erlangen. Beginning with the first entry in the randomization list, the staff of the pharmacy packs medication kits for one patient each. Every kit is labelled by a consecutive ID number and contains a sealed opaque envelope revealing the information about the medication. A pre-defined number of kits are packed into a package and are sent to the trial sites. The medical staff in the trial sites is compelled to take the set with the lowest identification number ever. The identification number of the trial drug package has to be linked to a patient identification number provided on the patient identification list. Within 24 h (working day) after the randomization the patient is registered in the database by the Clinical Trial Centre Leipzig.

### Study procedures

The trial drugs (either VPA or LEV) shall be administered in one infusion over 10 min. Both drugs are provided in identical colourless clear fluids of 50 ml volume. The formulation includes the maximum possible dosage of LEV 4.5 g and VPA 3 g. The entire volume shall always be transferred to the syringe for direct use, while the perfusor running rate depends on the (estimated) body weight. The dosages for the patients are: LEV 45 mg/kg and VPA 30 mg/kg. Patients with a weight of 100 kg and higher will receive the entire 50 ml of the infusion. Due to the formulated strengths, the infusion times are identical for LEV and VPA in order to maintain blinding. The drugs are manufactured, packaged and labelled by the Department of Pharmacy of the University Hospital Erlangen. After the initiation of infusion the patients’ clinical state will be evaluated after 15, 30 and 60 min. In patients with NCSE, EEG recording will be continued until the end of 60 min. The intervention is deemed successful if: 1) convulsive seizures or minimal rhythmic motor activity and/or EEG signs of NCSE according to the Salzburg criteria cease [13] during 15 min after the start of infusion AND 2) ictal activity does not recur within the following 45 min. Blood samples will be obtained for lab analysis including the plasma levels of both trial drugs before and 60 min after the initiation of infusion. Patients as well as the treating physicians are blinded for at least 15 and a maximum of 60 min after the initiation of infusion. The infusion must be stopped immediately if there are any clinical signs of an allergic reaction or if the patient experiences a medical emergency that necessitates the discontinuation or unblinding of the treatment. The infusion must also be stopped if the patient withdraws his/her consent to participate. Unblinding is required if initially ceased ictal activity recurs at any time between 15 and 60 min after the start of infusion.

After the intervention, patients will be followed until the day of discharge or day 30. Data will be obtained about complications of the disease and the hospital treatment including the rate of recurrence of seizures after successful intervention, the rate of SE-related ventilation, the incidence of infections and also data to assess the safety profile of both drugs. Adverse events will be recorded until the end of the study.

All persons participating in the conduct of the study (sponsor, authorized representative of the sponsor, investigators, etc.) commit themselves to observe the Declaration of Helsinki of the World Medical Association (in its current version, [21]), as well as all pertinent national laws and the ICH guidelines for Good Clinical Practice (GCP) ICH E6(R2) (EMA/CHMP/ICH/135/1995) issued in June 2017 [22].

**Fig. 1:**
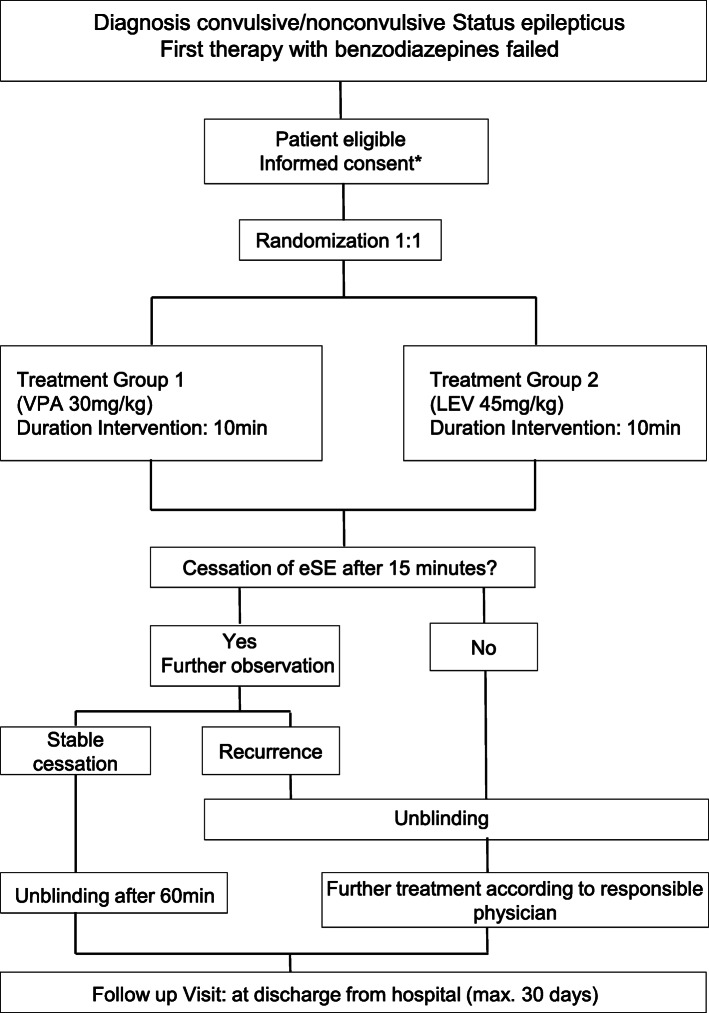
Flow chart detailing study procedures of enrollment, intervention and follow up. * procedures follow the paragraph 41 of the German Medicinal Products Act for the inclusion of persons who are not able to provide informed consent, inclusion procedure in strict adherence to the urgency of treatment eSE, established status epilepticus; VPA, valproate; LEV, levetiracetam

The intervention scheme is shown in Fig. 1, the visit schedule with all corresponding assessments is given in Table 1.

**Table 1:** Visit schedule with all corresponding assessments

Visit	Screening	V1					V2^a^
		Initiation of infusion				During 24 h after T0	Further Hospital stay
		T0	T15	T30	T60		
**Time**		0	15 min	30 min	60 min	24 h	
Eligibility criteria	X						
Informed consent^b^	X						
Randomization	X						
Medical/Medication History	X						
CP-Monitoring^c^		Continuously					
EEG^d^	X^4^	Continuously^4^					
Blood analysis^e^	X				X		
VPA **or** LEV infusion		X					
GCS^f^	X		X	X	X		X
Neurological exam	X				X		X
mRS/BI^g^	X^h^						X
Home care	X^h^						X^i^
Adverse events		X					
Recurrence seizures/SE		Recorded any time until discharge					
Clinical data^j^		X					

*VP*A Valproate, *LEV* Levetiracetam, *(NC)SE* (nonconvulsive) status epilepticus

^a^Follow-up-visit, at day of discharge or day 30

^b^by legal or authorised representative **or** according to §41 AMG (1)

^c^cardiopulmonary monitoring

^d^electroencephalography, only in patients with NCSE

^e^complete blood count, liver, kidney function, sodium, level of VPA, level of LEV

^f^Glasgow Coma Scale

^g^modified Rankin Scale/Barthel Index

^h^premorbid state by patient (retrospective) **or** relatives

^i^ after hospital stay (if applicable)

^j^clinical data about the hospital stay, i.e. infections with i.v. antibiotics, special medications, initiation of invasive/noninvasive ventilation, recorded on day of discharge/day 30

#### EEG recording

As the study aims to reflect the clinical routine of the diagnosis and treatment of SE, there are no predefined technical standards of the EEG recording including number and placement of electrodes. The recording has to cover the whole observation period of 60 min after the start of intervention. It is not possible to replace the EEG after the initiation of infusion. NCSE will be diagnosed according to the Salzburg criteria [13]:

EEG patterns have to be continuously present for at least 10 s and the whole EEG recording should be abnormal AND epileptiform discharges > 2.5 Hz OR epileptiform discharges ≤2.5 Hz with fluctuation or typical spatiotemporal evolution or subtle clinical ictal phenomenon OR rhythmic delta/theta activity (> 0.5 Hz) are detected.

15 min after the start of infusion the study physician will check if there is an absence of ictal patterns consistent with NCSE. In case of cessation of NCSE, EEG signs will be checked frequently in the following 45 min to record recurrence of ictal activity. In case of persistence of NCSE, EEG signs will be checked for cessation of ictal activity during 60 min after the start of infusion.

EEG recordings will be reviewed by two independent experts who are blinded to the treatment arm and to clinical data (HH and FR). The central procedure for review is established to ensure consistency among the hospitals in the diagnosis and the determination of cessation of SE. All investigators will be trained in evaluation of EEG data before the start of enrolment.

#### Laboratory markers

Laboratory assessments for the levels of VPA and LEV, blood count, sodium, liver and kidney function are done before and 60 min after the start of infusion. In addition, the blood glucose has to be checked before the initiation of infusion as hypoglycemia is a criterion of exclusion of the patient.

#### Clinical assessments

All the study participants are offered standard care of SE-treatment including a cardiopulmonary monitoring comprising assessment of heart rate, oxygen saturation and blood pressure. Before the initiation of infusion, the type of SE is assessed: generalized, focal with impaired consciousness or focal without impaired consciousness, NCSE with coma or without coma. During the observation period of 60 min, the vigilance status is checked using the Glasgow Coma Scale [23] at the start of infusion and 15, 30 and 60 min later. In addition, a brief neurological exam including orientation, eye movements, muscle strength testing of both arms and legs, sensation and language is performed before the start of infusion and 60 min later. The persistence or cessation of clinical signs of SE such as convulsions or minimal rhythmic motor activity is monitored beginning from 15 min after the start of infusion and continuously for the following 45 min.

#### Blinding

Study participants and study staff are both blinded to the trial drugs. In case of persistence of SE at 15 min after the initiation of infusion the medication is unblinded and the subsequent procedure is to be executed at the discretion of the treating physician. In case of cessation of SE at 15 min after the start of infusion, the medication will be unblinded at the time of recurrence of ictal activity during the following 45 min. If ictal activity is controlled for the duration of observation, the medication will be unblinded at 60 min after the start of infusion. This is done by opening the sealed envelope in the medical kit.

#### Premorbid state and follow-up-visit

The evaluate the consequences of the disorder on functional outcome the modified Rankin Scale [24] and the Barthel Index [25] will be obtained. After inclusion, the premorbid state according to both scales will be estimated retrospectively by an interview with the patient or a relative. On the day of discharge or on day 30 both scales will be determined prospectively.

### Specific objectives

Primary goal of ToSEE is to generate evidence for the treatment of established SE in an elderly population. Primary endpoint is the effectiveness of intravenous VPA or LEV to terminate BRSE and maintain control of epileptic activity up to 60 min after initiation of the study intervention. The secondary endpoints are to assess the safety profile of VPA and LEV in elderly patients with BRSE, other measures of efficacy, and to collect observational data about SE in the elderly. They are given in Table 2.

**Table 2:** Secondary endpoints

Efficacy	
• Time from initiation of study intervention to cessation of eSE within 60 min	
• Neurological status (including vigilance) 60 min after initiation of intervention	
• Difference of blood levels of VPA and LEV before and 60 min after initiation of intervention	
• Recurrence of seizures or noncovulsive/convulsive SE after initially successful intervention	
• For patients who failed the primary endpoint, number of patients in whom SE ceased during 60 min after initiation of intervention according to the treating physician	
• For NCSE patients who failed the primary endpoint, time to first cessation, as verified by EEG	
• Number of patients with SE- associated ventilation until hospital discharge	
• Functional outcome at discharge, defined by Barthel Index and modified Rankin Scale	
Safety	
• Mortality	
• Need for any emergency medication (different from allocated study drug) during 60 min after initiation of study intervention	
• Need for ventilation (noninvasive/ invasive) during 60 min after initiation of intervention	
• Intrahospital complications	
° Incidence of delirium as diagnoses by the treating physician	
° Infections requiring intravenous administration of anti-infectives	
° Adverse events related to infusion/ subsequent therapy with antiepileptic drug (sedation, dizziness, nausea, vomiting, thrombocytopenia, leukopenia, hypotension, new elevation of liver enzymes, hyperammonaemia, acute new liver or pancreatic damage, tremor, psychiatric abnormalities)	

### Data and safety monitoring plan

The information entered into the trial database by the investigator or an authorized member of the trial team is systematically checked for completeness, consistency and plausibility such that discrepancies can be dealt with at data entry. By on-site monitoring or central/statistical monitoring, the monitor or the data manager at the Clinical Trial Centre Leipzig may create a manual query for discrepancies that are identified later. All electronic case report file (eCRF)- pages with queries are marked in the system and a report with all queries listed is available. The site staff is responsible for data correction. At the end of the study, once the database has been declared complete and accurate, the database will be locked. Thereafter, any changes to the database are possible only by joint written agreement between sponsor/sponsors authorized representative/ coordinating investigator, biometrician and data manager.

To guarantee the patient’s safety, an independent Data Monitoring Committee (DMC) will meet periodically to perform a review and an evaluation of the study data regarding the safety of the study intervention, the integrity and validity of the data, the appropriate study conduct and the study progress. The DMC consists of three individual experts who are not involved in the ToSEE study activities and who have no conflicts of interests with any of the participating organizations. They have sufficient combined expertise in the medical disciplines.

### Statistical analysis

Primary analysis is based on the full-analysis set of all patients who received study medication according to ICH E-9. The primary endpoint will be analyzed by means of a generalized mixed linear model with binomial link function incorporating randomization strata as fixed and centers as random effects. Odds ratios are calculated as effect sizes incl. 95% confidence interval (CI). In secondary analysis, the cessation rates are tested by chi-squared test. Cessation rates and their difference with 95% CI following Wilson [26, 27] are calculated. Other event rates are estimated with 95% CI (Wilson) and possibly are compared by chi-squared test without continuity correction and by mid-p test if expected numbers are too small, respectively. Time-to-cessation is analyzed by time-to-event methods (Kaplan-Meier estimates and logrank test). Latency between start of SE and 1) administration of the last benzodiazepines before the start of the study medication and 2) administration of the study medication is analyzed by descriptive statistics for convulsive SE and NCSE separately. An adjustment of the primary analysis for these covariates will be considered. (Generalized) mixed linear models are built for the other endpoints (e.g. questionnaire scores) as well for longitudinal analyses similar to the primary endpoint. In secondary analysis, the inclusion of the Status Epilepticus Severity Score (STESS) [28] into multiple models for several endpoints is envisaged.

## Discussion

Early and effective treatment of SE is associated with lower morbidity and mortality. After the first stage, consisting in the application of benzodiazepines, the choice of medication is uncertain in the elderly, because sufficient evidence is lacking. If generalized convulsive SE cannot be terminated at stage II, treatment of stage III which requires mechanical ventilation, may constitute an additional risk of its own, especially for older people [29]. The multicenter RCT “Established Status Epilepticus Treatment Trial - ESETT” (launched in 10/2015) enrolled patients over 2 years with no upper age limit and compared VPA, LEV and FPHT as the treatment for benzodiazepine refractory convulsive SE [9]. Also first results of a German observational study about SE-treatment in adults are published which focus on the SE termination at stage I [6]. In consideration of the current evidence the revised German guidelines (forthcoming in 2020 [30]) recommend FPHT, LEV or VPA as first line treatment and phenytoin, phenobarbital or lacosamide as second line for the treatment of BRSE. No new age specific evidence regarding treatment of SE in elderly people can be derived from these publications. Furthermore, most of the past studies and narrative reviews exclusively focus on the treatment of convulsive SE [15, 31]. In 1998 Treiman and colleagues (1998) published data about the effect of lorazepam, phenobarbital, diazepam plus phenytoin and phenytoin alone in the initial treatment of overt and subtle types of generalized SE [32]. 226 out of 518 evaluable patients were older than 65 years old in this study, 69 of them had subtle generalized SE. A subgroup analysis revealed different effects of the drugs according to the type of SE with phenobarbital being superior to lorazepam in the treatment of the subtle ones [33]. Another trial that compared lacosamide and FPHT for the treatment of nonconvulsive seizures and NCSE had to be stopped because of funding issues and slow enrollment [34]. A total of 74 subjects could be enrolled. Based on these data so far there are no randomized trials that suggest drugs and therapy regimes for the treatment of NCSE.

ToSEE is the first randomized study capable of providing evidence for the SE-therapy in the elderly. Different problems that may have yet precluded the initiation of a large trial as the process of informed consent and the establishment of a blinded intervention not causing an unethical delay of treatment have been taken into account. All types of SE shall be considered. The study also shall collect information about SE including further therapy strategies, complications of the disease and outcome aspects in the short term.

### Study details

The University of Leipzig, Leipzig, Germany is the sponsor of the study. The study will be initiated in August 2020.

## Supplementary Information

**Additional file 1.** Trial protocol Treatment of Established Status Epilepticus in the Elderly – a prospective, randomized, double-blind comparative effectiveness trial ToSEE, Date: 2020-05-18, Version: final 4.0.

**Additional file 2.** SPIRIT Checklist: Treatment of Established Status Epilepticus in the Eldery – a prospective multicentre double-blind comparative effectiveness trial (ToSEE, EudraCT-No.: 2018–003917-16).

## Data Availability

Data (as case report file, patient consent form etc.) are available from the corresponding author on reasonable request.

## Abbreviations

BI: Barthel Index
BRSE: Benzodiazepine refractory status epilepticus
CHMP: Committee for Human Medicinal Products
DMC: Data Monitoring Committee
eCRF: Electronical case report file
EEG: Electroencephalography
EMA: European Medicines Agency
GCP: Good Clinical Practice
GCS: Glasgow Coma Scale
ICH: International Council for Harmonisation
i.v.: Intravenous
LEV: Levetiracetam
mRS: Modified Rankin Scale
NCSE: Nonconvulsive status epilepticus
OR: Odds ratio
VPA: Valproate
STESS: Status Epilepticus Severity Score
ToSEE: Treatment of Established Status Epilepticus in the Elderly

## Footnotes

1 benzodiazepine refractory status epilepticus is synonym for the term established status epilepticus which is used in the study protocol and in Figure 1
