# Supplementary material for: Treatment of established status epilepticus in the elderly - a study protocol for a prospective multicenter double-blind comparative effectiveness trial (ToSEE)
Source: BMC Neurol. 2020 Dec 3;20:438. doi: 10.1186/s12883-020-02001-x (PMC7713039; doi:10.1186/s12883-020-02001-x)
Supplement: Supplementary file 1 — Additional file 1. Trial protocol Treatment of Established Status Epilepticus in the Elderly – a prospective, randomized, double-blind comparative effectiveness trial ToSEE, Date: 2020-05-18, Version: final 4.0. [file 12883_2020_2001_MOESM1_ESM.pdf]

Trial Protocol

Treatment of Established Status Epilepticus in the Elderly - a prospective, randomized, double-blind comparative effectiveness trial

ToSEE

Gefördert vom Bundesministerium für Bildung und Forschung

Förderkennzeichen: 01GL1804

Coordinating investigator

Prof. Dr. med. Joseph Claßen

Klinik und Poliklinik für Neurologie

Universitätsklinikum Leipzig

Liebigstraße 20, 04103 Leipig, Germany

Biometry

Dr. rer. nat. Meinhard Mende

Zentrum für Klinische Studien Leipzig

Universität Leipzig

Härtelstraße 16-18, 04107 Leipzig, Germany

Sponsor

University of Leipzig,
Ritterstr. 26, 04109 Leipzig

Date: 2020-05-18

Version: final 4.0

EudraCT-No.: 2018-003917-16

DRKS-No.: not yet available

Table of Contents

SEITE

GENERAL INFORMATION 4

Responsible Parties 4
Synopsis (English) 6
Synopse (Deutsch) 8
Schedule of Assessments and Procedures 12
Flow Chart 13

1 RATIONALE 14

1.1 Medical Background 14
1.2 Rationale 15
1.3 Risk-Benefit Considerations 15

2 OBJECTIVES 17

2.1 Primary Objective 17
2.2 Secondary Objectives 17

3 TRIAL DESIGN AND DESCRIPTION 17

3.1 Trial Design 17
3.2 Requirements at the Trial Sites regarding Personnel and Equipment 18
3.3 Trial Sites and Number of Trial Subjects 19
3.4 Expected Duration of Trial 19
3.5 Premature Termination of the Trial 19

4 TRIAL SUBJECTS 20

4.1 Inclusion Criteria 20
4.2 Exclusion Criteria 21
4.3 Justification for the Inclusion of vulnerable Populations 22
4.4 Participation in more than one Clinical Trial 24
4.5 Statement on the Inclusion of Dependent Individuals 24
4.6 Rationale for Gender Distribution 24

5 INVESTIGATIONAL PRODUCT 24

5.1 Trial Drugs 24
5.2 Packaging and Labelling of the Trial Drug 25
5.3 Storage and Handling Requirements 25
5.4 Drug Accountability 25
5.5 Administration of the Study Drugs 26
5.6 Blinding and Unblinding 29

6 INDIVIDUAL TRIAL PROCEDURES 29

6.1 Patient Information and Informed Consent 29
6.2 Enrolment in the Trial 30
6.3 Description of the Treatment Procedures 32
6.4 Premature Termination of the Trial 35
6.5 Plan for Further Treatment 36

7 ADVERSE EVENTS (AE/SAE) 36

7.1 Adverse Events (AE) 36
7.2 Safety Analysis 37
7.3 Concomitant Diseases 37
7.4 Serious Adverse Events/Serious Adverse Reactions (SAE/SAR) 38
7.5 Periodic Reports 39

7.6 Suspected Unexpected Serious Adverse Reactions (SUSAR) 40
7.7 Other Safety Relevant Issues 40
7.8 Therapeutic Procedures 41
7.9 Dealing with Pregnancy 41

8 BIOMETRY 41

8.1 Biometrical Aspects of the Trial Design 41
8.2 End Points 42
8.3 Statistical Description of the trial hypothesis 44
8.4 Sample Size Discussion 44
8.5 Statistical Methods 45
8.6 Statistical Monitoring 45
8.7 Interim Analysis 46
8.8 Final Analysis 46

9 ETHICAL, LEGAL AND ADMINISTRATIVE ASPECTS 46

9.1 GCP-Statement 46
9.2 Initial Submission 46
9.3 Protocol Amendments 47

10 DOCUMENTATION 47

10.1 General information and Access Rights 47
10.2 Patient File and Source Data 48
10.3 Data Management 48
10.4 Archiving 49

11 REFERENCE EVALUATIONS 49

12 SUPERVISION OF THE CLINICAL TRIAL 49

12.1 Access to Source Data 49
12.2 Monitoring 50
12.3 Audits 50
12.4 Inspections 50
12.5 Independent Supervision of the Trial 51
12.6 Data protection and Confidentiality 52
12.7 Declaration regarding Data Protection 53
12.8 Declaration regarding the Pseudonymized Transfer of Personal Data 53
12.9 Anonymisation of Data after the end of Archiving 53

13 ADMINISTRATIVE AGREEMENTS 53

13.1 Adherence to the Protocol 53
13.2 Funding and Insurance 54
13.3 Notification of the Local Authorities 54
13.4 Publication Policy and Registration 54
13.5 Data Sharing Statement 55

14 PROTOCOL SIGNATURES 56

15 PROTOCOL AGREEMENT 57

16 APPENDIX 58

16.1 Classification of Adverse Events 58
16.2 Acronyms 60
16.1 Template trial protocol 61

17 REFERENCES 62

GENERAL INFORMATION

Responsible Parties

Sponsor<br>(according to German Medicinal Products Act)	**University of Leipzig**<br>Ritterstr. 26, 04109 Leipzig<br>Authorised representative of the sponsor:<br>Prof. Dr. med. Joseph Claßen
Coordinating investigator	Prof. Dr. med. Joseph Claßen<br>Klinik und Poliklinik für Neurologie<br>Universitätsklinikum Leipzig<br>Liebigstraße 20, 04103 Leipzig<br>Tel: ++49 (0)341/9724200; Fax: ++49 (0)341/9724209<br>Joseph.Claßen@medizin.uni-leipzig.de
Deputy of Coordinating investigator	Prof. Dr. med. Dominik Michalski<br>Klinik und Poliklinik für Neurologie<br>Universitätsklinikum Leipzig<br>Liebigstraße 20, 04103 Leipzig<br>Tel: ++49 (0)341/97339; Fax: ++49 (0)341/9724209
Medical consultants<br>(EEG Reference Board)	Prof. Dr. med. Felix Rosenow, MHBA<br>Epilepsiezentrum Frankfurt Rhein-Main<br>Zentrum der Neurologie und Neurochirurgie<br>Universitätsklinikum Frankfurt<br>Goethe-Universität<br>Schleusenweg 2-16, 60528 Frankfurt am Main<br>Tel: ++49 (0)69/63017466; Fax: ++49 (0)69/630185065<br>rosenow@med.uni-frankfurt.de<br><br>Prof. Dr. med. Hajo Hamer, MHBA<br>Epilepsiezentrum in der Neurologischen Klinik<br>Universitätsklinikum Erlangen<br>Schwabachanlage 6, 91054 Erlangen<br>Tel:++49(0)9131/85-39116;Fax: ++49(0)9131/85-36469<br>Hajo.Hamer@uk-erlangen.de
Project Management	Dr. rer. nat. Anett Schmiedeknecht<br>Zentrum für klinische Studien Leipzig<br>Universität Leipzig<br>Medizinische Fakultät<br>Härtelstraße 16-18, 04107 Leipzig<br>Tel: ++49 (0)341/9716256; Fax: ++49 (0)341/9716189<br>Anett.Schmiedeknecht@zks.uni-leipzig.de
Pharmacovigilance	Madlen Dörschmann<br>Zentrum für klinische Studien Leipzig<br>Universität Leipzig<br>Medizinische Fakultät<br>Härtelstraße 16-18, 04107 Leipzig<br>Tel: ++49 (0)341/9716129<br>Fax: ++49 (0)341/9716278<br>pharmacovigilance@zks.uni-leipzig.de
Data Management	Marc Viehweg<br>Stefanie Lehmann<br>Zentrum für klinische Studien Leipzig<br>Universität Leipzig<br>Medizinische Fakultät<br>Härtelstraße 16-18, 04107 Leipzig

	Tel: ++49 (0)341/9716262<br>Marc.Viehweg@zks.uni-leipzig.de<br>Tel: ++49 (0)341/9716263<br>Stefanie.Lehmann@zks.uni-leipzig.de
Biometry	Dr. rer. nat. Meinhard Mende<br>Zentrum für klinische Studien Leipzig<br>Universität Leipzig<br>Medizinische Fakultät<br>Härtelstraße 16-18, 04107 Leipzig<br>Tel: ++49 (0)341/9715770<br>Meinhard.Mende@zks-uni-leipzig.de
Monitoring	Monika Rohwedder<br>Dr. rer. nat. Reinhild Schnabel<br>Dr. rer. nat. Susanne Melzer<br>Zentrum für klinische Studien Leipzig<br>Universität Leipzig<br>Medizinische Fakultät<br>Härtelstraße 16-18, 04107 Leipzig<br>Tel: ++49 (0)341/9716281<br>Monika.Rohwedder@zks.uni-leipzig.de<br>Tel: ++49 (0)341/9716258<br>Reinhild.Schnabel@zks.uni-leipzig.de<br>Tel: ++49 (0)341/9716317<br>Susanne.Melzer@zks.uni-leipzig.de
Funding	BMBF Projekträger im DLR<br>Frau Dr. Michaela Fersch<br>Heinrich-Konen-Str. 1<br>D-53277 Bonn<br>Förderkennzeichen: 01GL1804
Data Monitoring Committee	Prof. Dr. med. Bernhard Steinhoff<br>Epilepsiezentrum Kork<br>Landstraße 1, 77694 Kehl-Kork<br>Tel: ++49 (0)7851/842250; Fax: ++49 (0)7851/842555<br>BSteinhoff@epilepsiezentrum.de<br><br>Dr. med. Christian Brandt<br>Epilepsiezentrum Bethel<br>Krankenhaus Mara gGmbH<br>v. Bodelschwinghsche Stiftungen Bethel<br>Maraweg 21, 33617 Bielefeld<br>Tel: ++49 (0)521/77278804;<br>Fax: ++49 (0)521/77278809<br>Christian.Brandt@mara.de<br><br>Frau Inga Steinbrenner<br>Universitätsklinikum Freiburg<br>Studienzentrum<br>Elsässer Straße 2<br>79110 Freiburg<br>Telefon: ++49 (0)761 270-77107<br>Telefax: ++49 (0)761 270-73730

Synopsis (English)

Title of the trial	Treatment of Established Status Epilepticus in the Elderly - a prospective, randomized, double-blind comparative effectiveness trial
Acronym	ToSEE
Indication	Convulsive or nonconvulsive benzodiazepine-resistant (established) status epilepticus (eSE) in people 65 years or older
Primary objective of the trial/ primary endpoint	Primary goal is to generate evidence for the treatment of established SE in an elderly population.<br><br>Primary endpoint is the effectiveness of intravenous valproate (VPA) or levetiracetam (LEV) to terminate eSE and maintain control of epileptic activity up to 60 minutes after initiation of the trial intervention.
Secondary objectives of the trial/secondary endpoints	Secondary goals are to assess the safety profile of VPA and LEV in elderly patients with eSE, and to collect observational data about eSE.<br><br><u>Key secondary endpoints:</u><br>• Time from initiation of trial intervention to cessation of eSE within 60 minutes<br>• Neurological status (including vigilance) 60 minutes after initiation of intervention<br>• Difference of blood levels of VPA and LEV before and 60 minutes after initiation of intervention<br>• Recurrence of seizures or nonconvulsive/ convulsive SE after initially successful intervention<br>• For patients who failed the primary endpoint, number of patients in whom SE cessated during 60 minutes after initiation of intervention according to the treating physician<br>• For NCSE patients who failed the primary endpoint, time to first cessation, as verified by EEG<br>• Number of patients with SE-associated ventilation until hospital discharge<br>• Functional outcome at discharge, defined by Barthel Index (BI) and modified Rankin Scale (mRS)<br><br><u>Assessment of safety:</u><br>• Mortality<br>• Need for any emergency medication (different from allocated study drug) during 60 minutes after initiation of study intervention<br>• Need for ventilation (noninvasive/ invasive) during 60 minutes after initiation of intervention<br>• Intrahospital complications<br>o Incidence of delirium as diagnosed by the treating physician<br>o Infections requiring intravenous administration of anti-infectives<br>o Adverse events related to infusion/subsequent therapy with antiepileptic drug (sedation, dizziness, nausea, vomiting, thrombocytopenia, leukopenia,

	hypotension, new elevation of liver enzymes, hyperammonaemia, acute new liver failure or pancreatic damage, tremor, psychiatric abnormalities)
Trial design	Multicenter prospective, controlled, double-blind, randomized, comparative effectiveness phase IV-trial with two treatment arms
Trial population	Key inclusion criteria:<br><br>Adult patients ≥ 65 years old with ongoing convulsive SE (generalized CSE/focal CSE with impaired consciousness/focal CSE without impaired consciousness), as defined by a seizure lasting ≥ 5 minutes or 2 or more convulsive seizures without full recovery of consciousness ≥ 5 minutes, or nonconvulsive SE (NCSE with coma/ NCSE without coma) defined as ongoing EEG patterns consistent with definite or possible NCSE according to the Salzburg criteria (Leitinger et al. 2016), or clinically defined NCSE non-responding to treatment with AT LEAST<br>• Lorazepam 2 mg (i.v.)<br>• Midazolam 5 mg (i.v., buccal, intranasal, i.m.)<br>• Diazepam 5 mg (i.v., rectal)<br>• Clonazepam 1 mg (i.v.)<br><br>Key exclusion criteria:<br>• Treatment of SE with other antiepileptic drugs/sedatives before enrollment<br>• **Intravenous** application of VPA or LEV in the last 24 hours before enrollment.<br>• Known or suspected severe liver or pancreatic disease (alcohol addiction, known liver cirrhosis or familial liver diseases, clinical signs of severe liver disease such as ascites, jaundice)<br>• Known concomitant treatment with one or several of the following medications: phenobarbital, phenytoin, carbamazepine, carbapenem antibiotics, rifampicin, erythromycin, cimetidine, primidone, mefloquine, fluoxetine, felbamat, lopinavir, ritonavir<br>• Known coagulopathy (anticoagulants allowed)<br>• Known porphyria, mitochondriopathy and urea cycle disorders<br>• Known severe kidney disease (GFR < 30ml/min)<br>• Known insulin dependent diabetes mellitus<br>• Hypoglycemia (< 3.3 mmol/l)<br>• Estimated weight < 45kg.<br>• Need for acute neurosurgical treatment.<br>• Known cardiopulmonary resuscitation within the last 7 days before enrollment<br>• Known hypersensitivity against VPA or LEV<br>• Known participation in other interventional trials<br>• Known former participation in this trial
Sample size	To be allocated to trial n=477<br>To be analyzed n=454
Therapy	Patients will be randomised 1:1 into two treatment arms: 30mg/kg VPA i.v. or 45mg/kg LEV i.v., duration of infusion (intervention) 10min, duration of blinded phase max. 60min, unblinding to ensure appropriate further therapy according to success/failure of study intervention

Biometry	Efficacy: Generalised linear mixed model including randomisation strata as covariate and trial site as random intercept, estimation of odds ratio and rate difference with 95% confidence interval (CI).<br>Description of the primary efficacy analysis and population:<br>Full analysis set (FAS): all patients who received the trial medication will be analysed as randomized (intention-to-treat principle)<br>Safety: Frequencies with 95% CI of complication rates and (S)AEs<br>Secondary endpoints: Event rates (incl. 95% CI), chi² test, time to cessation of SE by time-to-event methods, (generalized) mixed linear models for longitudinal analyses as well for the other endpoints (e.g. questionnaire scores) similar to the primary endpoint.
Trial Duration	Duration per patient:<br>Duration of intervention: 10 minutes<br>Duration of the trial: until discharge from hospital, maximum 30 days after enrollment<br>Duration of the trial:<br>First patient in to last patient out (months): 48<br>Recruitment period (months): 47<br>Duration of the entire trial, including preparation and analysis (months): 60

Synopse (Deutsch)

Studientitel	Therapie des Benzodiazepin-resistenten Status Epilepticus in der älteren Bevölkerung
Akronym	ToSEE
Indikation	Konvulsiver oder nonkonvulsiver Benzodiazepin-resistenter („etablierter“) Status epilepticus (eSE) bei Patienten, die 65 Jahre oder älter sind
Primäres Studienziel / primärer Endpunkt	Primäres Studienziel ist es Evidenz zu schaffen für die Therapie des etablierten Status epilepticus in der älteren Bevölkerung.<br>Primärer Endpunkt ist die Beendigung des eSE und der Erhalt der Kontrolle über die epileptische Anfallsaktivität für mindestens 60 Minuten nach Beginn der Studienintervention mit intravenöser Gabe von Valproat (VPA) oder Levetiracetam (LEV).
Sekundäre Studienziele / Endpunkte	Sekundäres Studienziel ist zum einen die Untersuchung des Sicherheitsprofils von VPA und LEV und zum anderen die Sammlung observationaler Daten über den eSE.<br><br>Sekundäre Haupt-Endpunkte:<br>• Zeit vom Beginn der Studienintervention bis zum Durchbrechen des eSE<br>• Klinisch-neurologischer Befund (inklusive Bewusstseinszustand) 60 Minuten nach Beginn der Studienintervention<br>• Differenz der Medikamentenspiegel von VPA und LEV vor und 60 Minuten nach Beginn der Intervention

	• Wiederauftreten von konvulsiven oder nonkonvulsiven Anfällen/SE nach erfolgreicher Intervention<br>• Bei fehlgeschlagener Intervention: Anzahl der Patienten, bei denen laut Einschätzung des behandelnden Arztes der SE innerhalb von 60 Minuten nach Start der Intervention beendet wurde<br>• Zeit bis zum ersten EEG-gestützten Nachweis des beendeten nonkonvulsiven SE nach fehlgeschlagener Intervention<br>• Anzahl an Patienten mit SE-assoziierter Beatmung bis zur Entlassung aus der Klinik<br>• Funktioneller Status zum Zeitpunkt der Entlassung, definiert über Barthel Index (BI) und modified Rankin Scale (mRS)<br><br>Sicherheits-Endpunkte:<br>• Mortalität<br>• Einsatz jeglicher Notfallmedikamente (unterschiedlich vom Studienmedikament) innerhalb von 60 Minuten nach Beginn der Intervention<br>• Notwendigkeit der Beatmung (nicht invasiv/invasiv) innerhalb von 60 Minuten nach Beginn der Intervention<br>• Komplikationen im stationären Aufenthalt:<br>o Inzidenz des Delirs, Diagnose gestellt vom behandelnden Arzt<br>o Infektionen, die eine intravenöse Antiinfektiva-Therapie erfordern<br>o Unerwünschte Ereignisse im Zusammenhang mit der Infusion der Prüfmedikation bzw. der weiterführenden Therapie mit dem antikonvulsiven Medikament (Sedierung, Benommenheit, Übelkeit, Erbrechen, Thrombozytopenie, Leukopenie, Blutdruckabfall, neue Leberwerterhöhung, Hyperammonämie, akute neue Leber- oder Pankreasschädigung, Tremor, psychiatrische Auffälligkeiten)
Studiendesign	Multizentrische, prospektive, kontrollierte, doppel-blinde, randomisierte Studie zum Phase-IV-Vergleich zweier Behandlungsarme
Studienpopulation	Einschlusskriterien:<br><br>Erwachsene Patienten ≥ 65 Jahre mit anhaltendem konvulsiven SE (Status generalisierter tonisch-klonischer Anfälle/ fokaler SE mit oder ohne Einschränkung des Bewusstseins) für ≥ 5 Minuten oder 2 oder mehr konvulsiven Anfällen ohne Wiedererlangen des Bewusstseins zwischen den Anfällen für ≥ 5 Minuten oder nonkonvulsiver SE (NCSE mit Koma/ NCSE ohne Koma) definiert als andauernde EEG-Veränderungen vereinbar mit möglichem/ sicherem NCSE entsprechend der Salzburger Kriterien (Leitinger et al. 2016) oder klinisch diagnostiziertem NCSE<br><br>ohne Therapieansprechen auf MINDESTENS<br>• Lorazepam 2 mg (i.v.)<br>• Midazolam 5 mg (i.v., bukkal, intranasal, i.m.)<br>• Diazepam 5 mg (i.v. rektal)<br>• Clonazepam 1 mg (i.v.)

	Ausschlusskriterien:<br>• Behandlung des SE mit anderen Antiepileptika/Sedativa vor Einschluss<br>• **Intravenöse** Applikation von VPA oder LEV innerhalb von 24 Stunden vor Einschluss<br>• Bekannte oder anzunehmende schwere Leber- oder Pankreaserkrankung (alkoholabhängige Patienten, bekannte Leberzirrhose oder fämiliäre Lebererkrankungen, klinische Zeichen einer schweren Lebererkankung, z.B. Aszites, Ikterus)<br>• Bekannte Begleitmedikation mit einem oder mehreren der folgenden Medikamente: Phenobarbital, Phenytoin, Carbamazepin, Carbapenem-Antibiotika, Rifampicin, Erythromycin, Cimetidin, Primidon, Mefloquin, Fluoxetin, Felbamat, Lopinavir, Ritonavir<br>• Bekannte Störung der Blutgerinnung (Antikoagulantien sind erlaubt)<br>• Bekannte Porphyrie, Mitochondropathie oder Störung des Harnstoffzyklus<br>• Bekannte höhergradige Nierenfunktionseinschränkung (GFR < 30 ml/min)<br>• Bekannter insulinpflichtiger Diabetes mellitus<br>• Hypoglykämie (< 3,3 mmol/l)<br>• Geschätztes Gewicht < 45 kg<br>• Notwendigkeit einer akuten neurochirurgisch- operativen Behandlung<br>• Bekannte kardiopulmonale Reanimation innerhalb von 7 Tagen vor Einschluss<br>• Bekannte Überempfindlichkeit gegenüber VPA oder LEV<br>• Bekannte Teilnahme an anderen Interventionsstudien<br>• Bekannte frühere Teilnahme an dieser Studie
Patientenzahl	Zu randomisieren n=477<br>Zu analysieren n=454
Therapie	Die Patienten werden 1:1 in zwei Behandlungsarme randomisiert, 30mg/kg VPA oder 45mg/kg LEV i.v., Dauer der Infusion (Intervention) 10 Minuten, Dauer der Verblindung maximal 60 Minuten, Entblindung zur Gewährleistung einer adäquaten weiterführenden Therapie entsprechend Erfolg oder Misserfolg der vorangegangenen Intervention
Biometrie	Wirksamkeit: Verallgemeinertes gemischt-lineares Modell mit Randomisations-Strata als Kovariaten und Studienzentrum als zufälliger Konstante; Schätzung des Odds Ratios und der Differenz der Häufigkeiten mit 95% Konfidenzintervall (CI).<br>Beschreibung der primären Wirksamkeitsanalyse und Population<br>Full analysis set (FAS): Alle Patienten, welche die Studienmedikation erhielten, werden wie randomisiert analysiert (intention-to-treat Prinzip)<br>Sicherheit: Häufigkeiten der Komplikationsraten and (S)AEs mit 95% CI<br>Sekundäre Endpunktes: Eventraten (inkl. 95% CI), Chi²-Test, Zeit bis Durchbrechen des SE: Kaplan-Meier-Schätzer und Cox-Regression, geschätztes Hazard Ratio inkl. 95% CI.<br>Kontinuierliche Variablen (inkl. Fragebögen-Scores): gemischt-lineare Modelle, Schätzung der mittleren Differenzen inkl. CI

	(Verallgemeinerte) gemischt-lineare Modelle werden für Längsschnittauswertungen angewandt.
Zeitplan	Dauer pro Patient:<br>Dauer der Intervention: 10 Minuten<br>Dauer der Studie: bis zur Entlassung aus dem Krankenhaus bzw. maximal 30 Tage nach Einschuss<br>Dauer der Studie:<br>First patient in to last patient out (Monate): 48<br>Rekrutierungszeitraum (Monate): 47<br>Dauer der gesamten Studie, einschließlich Vorbereitung und Analyse (Monate): 60

Schedule of Assessments and Procedures

The following table shows the proposed visit schedule with all corresponding assessments.

**Visit**	**Screening**	**V1**						**V2**[1]
		Initiation of infusion **T0**	**T15**	**T30**	**T60**	During **24h** after **T0**	Further Hospital stay	
**Time**		0	15 min	30 min	60 min	24 h		
Eligibility criteria	X							
Informed consent[2]	X							
Randomziation	X							
Medical/ Medication History	X							
CP-Monitoring[3]		Continuously						
EEG[4]	X[4]	Continuously[4]						
Blood analysis[5]	X				X			
VPA **or** LEV infusion		X						
GCS[6]	X		X	X	X			X
Neurological exam	X				X			X
mRS/BI[7]	X[8]							X
Home care	X[8]							X[9]
Adverse events		X						
Recurrence seizures/SE		Recorded any time until discharge						
Clinical data[10]		X						

[1]Follow-up-visit, at day of discharge or day 30
[2]by legal or authorised representative **or** according to §41 AMG (1)
[3]cardiopulmonary monitoring
[4]electroencephalography, only patients with NCSE
[5]complete blood count, liver, kidney function, sodium, level of VPA, level of LEV
[6]Glasgow Coma Scale
[7]modified Rankin Scale/Barthel Index
[8]premorbid state by patient (retrospective) **or** relatives
[9] after hospital stay (if applicable)
[10]clinical data about the hospital stay, i.e. infections with i.v. antibiotics, special medications, initiation and duration of invasive/noninvasive ventilation, recorded on day of discharge/day 30

Flow Chart

Figure 1

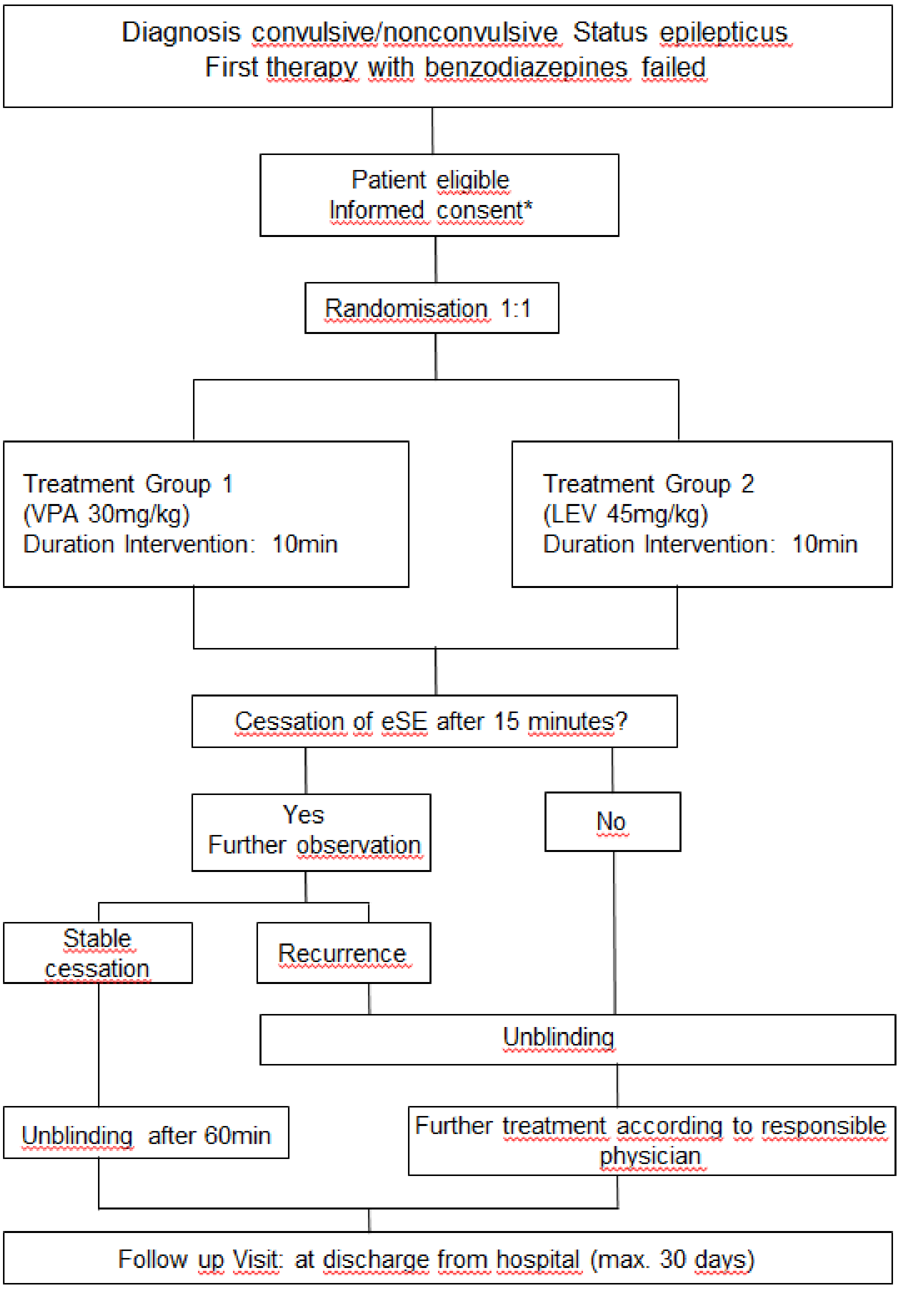

* detailed procedures are described in chapter 4.3

1 RATIONALE

1.1 Medical Background

Status epilepticus is after stroke the second most frequent neurological emergency condition. In the elderly and old population incidence of SE has its highest peak with 54.5 per 100.000 people over 60 years age in Germany (Knake et al. 2001) and case fatality rates are up to 50% in people over 80 years (DeLorenzo et al. 1992). Older people with SE are at special risk of suffering a poor outcome, partly because SE may be particularly challenging to treat, due to the nature of the causative brain injury, with stroke as main identified cause in 52% of SE cases (Legriel and Brophy 2016). Nonconvulsive SE (NCSE) is a frequent presentation of SE in older people. This condition is challenging to diagnose. On the one hand, its clinical presentation is highly variable and overlaps with other age-related neurological conditions such as delirium or dementia, on the other hand the diagnosis of NCSE mostly requires electroencephalographical validation. In the past, NCSE has been viewed as a relatively benign condition, because overt common and harmful side effects of convulsions as hyperthermia and acidosis are missing. However, recently it is increasingly recognized that NCSE incurs a risk of permanent neurological damage, especially in older people whose brain is more sensitive to prolonged epileptic activitiy (DeAssis et al. 2012). According to an epidemiological survey of Knake and colleagues the SE with impairment of consciousness (convulsive and nonconvulsive) covers the high majority of SE episodes with more than 80% of the cases (Knake et al. 2001). It particular seems representative in the elderly where the part of NCSE is assumed to be higher compared to younger adults.

As the pathomechanisms of SE unfold, and as longer duration of SE is associated with higher morbidity and mortality (Madzar et al. 2016), the treatment maxim of “time is brain” becomes appropriate not only for ischemic stroke but also for SE. Irrespective of the SE-subtype the recommended therapy follows a staged approach that classifys SE according to treatment and treatment response into early (I), established (II), refractory (III) and super-refractory (IV) SE (Trinka and Kälviäinen 2017). For adults there are several class I randomized clinical trials for stage I that support the recommendation for the benzodiazepines lorazepam (intravenous), midazolam (intravenous, buccal, intranasal, intramuscular), diazepam (intravenous, rectal) and clonazepam (intravenous) as effective first line therapy (Allredge et al. 2001; Silbergleit et al. 2012; Brigo et al. 2015). Following this therapy SE is successfully terminated in about two third of the cases. For the treatment of stage II the commonly used antiepileptic drugs phenytoin (PHE)/fosphenytoin, VPA, LEV, phenobarbital and lacosamide (LCM) are recommended. There are no high class RCTs (Randomized Controlled Trial) which would justify preferring one over the others. The current German guidelines (Leitlinie, Deutsche Gesellschaft für Neurologie) for the treatment of established SE in adults recommend PHE as first choice based on the the broad experience with this medication. However, as PHE has considerable cardiac arrhythmogenic potential treatment with PHE may not be advisable or even contraindiacted in elderly patients which exhibit high risks of developing cardiac arrhythmias. VPA, LEV and phenobarbital are listed as alternatives, lacosamide as further option in case of failure of previous therapies. For VPA, observations in a total of 860 SE patients are published, six randomized, but not double blinded RCTs provide an evidence Level B and indicate an overall response rate of about 70.9% with a good tolerability that also could be shown in critically ill patients who received dosages up to 40mg/kg (Trinka et al. 2015). The use of LEV has recently reported by a meta-analysis in which six RCTs with a total of 543 patients were included. LEV was compared to VPA, PHE and lorazepam and no significant difference in efficacy was found. (Chu et al. 2020)

Though phenobarbital has been widely used for treatment of SE and its efficacy has recently been confirmed again (Brigo et al. 2019) its clinical utility is limited by the central depressive effect with sedation, respiratory depression and by hypotension which are enhanced by prior administration of benzodiazepines (Trinka et al. 2015). The efficacy of LCM has been

investigated in several studies with different designs that indicate LCM as a more promising option for focal types of SE than for refractory or nonconvulsive types of SE. Reliable prospective data about the comparison to the recommended therapies are missing (Farrokh et al. 2019). Caution is recommended in patients with cardiac comorbidities because of the possible prolongation of the PR-interval in LCM (Leitlinie, Deutsche Gesellschaft für Neurologie; Strzelcyk et al. 2017).

Beyond these two stages anesthetics are used to control SE.

To face the lack of evidence the multicenter RCT “Established Status Epilepticus Treatment Trial - ESETT” (launch in 10/2015) enrolled patients over 2 years with no upper age limit and compared VPA, LEV and fosphenytoin as the treatment for established SE. Seventy of the enrolled 384 patients were older than 60 years old. Only convulsive SE has been considered in this trial. The anticonvulsant drugs each led to seizure cessation and improvement of consciousness by 60 minutes in approximately half of the patients with similar incidences of adverse events. (Kapur et al. 2019) Also first results of a German observational study about SE-treatment in adults are published which focus on the initial termination (Kellinghaus et al. 2019). No new evidence regarding treatment of SE in elderly people can be derived from this study. Another German observational study about outcome, economical issues and quality of life after SE is still recruiting (DRKS00008718).

1.2 Rationale

1.2.1 Hypothesis and Experimental Aspects of the Clinical Trial

ToSEE aims to identify an effective and safe therapy of benzodiazepine-resistant (“established”) status epilepticus in elderly and old patients. Among the 5 drugs commonly used for treatment of established SE, VPA and LEV deemed best suited to be considered for testing as they combine the best safety profile in elderly patients with documented, albeit insufficient, evidence of their efficacy. PHE was ruled out because of its known side-effects, in particular cardiac arrythmies and hypotension, with a special risk for people over 50 years age and with a pre-existing cardiac disease (Trinka et al. 2015). Lacosamide has been ruled out due to the lack of published prospective trials for this condition. Phenobarbital has been ruled out because of its sedative properties and respiratory depression.

Based on the existing literature we hypothesize a superiority in efficacy of VPA, the underlying facts are detailed in the chapter Biometry. Compared to the RCT ESETT (Kapur et al. 2019), used dosages of the intervention drugs are reduced to account for age dependent changes in pharmacokinetics including the decrease of the renal clearance (Contin et al. 2012) and the decrease in protein binding (DeAssis et al. 2012). With 30mg/Kg (maxium 3g) VPA and 45mg/Kg (maximum 4.5g) LEV the dosages are in line with current international guidelines (Leitlinie, Deutsche Gesellschaft für Neurologie; Glauser et al. 2016, Minicucci et al. 2020).

Due to the fact that ToSEE is a comparative effectiveness trial a control arm is not necessary.

ToSEE is the first randomized trial which shall deliver evidence for the SE- therapy in the elderly. It also shall collect information about this disease, its complications and outcome aspects. Even if no treatment difference can be proven, the full sample is desirable to obtain reliable estimates of each drug’s efficiency. Also potential superiority of one drug (possibly VPA) may be offset by a better safety profile of the other drug (LEV).

1.3 Risk-Benefit Considerations

Early and effective treatment of SE is associated with lower morbidity and mortality. After the first stage, consisting in the application of benzodiazepines, the choice of medication is uncertain, because sufficient evidence is lacking. If SE cannot be terminated at stage II,

treatment of stage III which requires mechanical ventilation, may constitute an additional risk of its own, especially for older people (Sutter et al. 2014).

According to the published literature (Kapur et al. 2019, Trinka and Kälviäinen 2017; Trinka et al. 2015) and the clinical experience VPA and LEV both appear to be safe and effective drugs in the treatment of SE. In the past, VPA was administered in effective doses from 15 to 45 mg/kg. Safety studies showed a generally low incidence of side effects (<10%; mainly thrombocytopenia and mild hypotension), that occurred independently of infusion rates. The most severe side-effects are hepatotoxicity and also encephalopathy, which has been observed to rarely follow hepatic dysfunction or hyperammonaemia (Trinka et al. 2014). LEV displays a low risk of side effects and drug interactions. Side effects included psychiatric disturbances, somnolence, fatigue and headache. The safety profile of the liquid formulation was comparable to the oral formulation. In single cases, thrombocytopenia, agitation, delirium and psychosis were described (Beuchat et al. 2018; Hwang et al. 2014).

Considering the lack of sufficient and robust scientific evidence, the life-threatening nature of the condition and the known poor outcome of older patients suffering from SE the conduction of this trial appears to be both needed and justified.

2 OBJECTIVES

2.1 Primary Objective

Primary goal is to generate evidence for the treatment of established SE in an elderly population.

2.2 Secondary Objectives

Secondary goals are to assess the safety profile of VPA and LEV in elderly patients with eSE, and to collect observational data about eSE.

Therefore, the following points will be evaluated:

- Time from initiation of intervention to cessation of eSE within 60 minutes
- Neurological status (including vigilance) 60 minutes after initiation of infusion
- Difference of blood levels of VPA and LEV before and 60 minutes after initiation of intervention
- Recurrence of seizures or nonconvulsive/ convulsive SE after initially successful intervention
- For patients who failed the primary endpoint, number of patients in whom SE cessated during 60 minutes after initiation of intervention according to the treating physician
- For NCSE patients who failed the primary endpoint, time to first cessation, as verified by EEG
- Number of patients with SE-associated ventilation until hospital discharge
- Functional outcome at discharge, defined by Barthel Index (BI) and modified Rankin Scale (mRS)
- Mortality
- Need for any emergency medication (different from allocated study drug) during 60 minutes after initiation of study intervention
- Need for ventilation (noninvasive/ invasive) during 60 minutes after initiation of intervention
- Intrahospital complications
    - Incidence of delirium as diagnosed by the treating physician
    - Infections requiring intravenous administration of anti-infectives

Adverse events possibly related to infusion/subsequent therapy with antiepileptic drug (sedation, dizziness, nausea, vomiting, thrombocytopenia, leukopenia, hypotension, new elevation of liver enzymes, hyperammonaemia, acute new liver failure or pancreatic damage, tremor, psychiatric abnormalities).

3 TRIAL DESIGN AND DESCRIPTION

3.1 Trial Design

ToSEE is a multicenter prospective, controlled, double-blind, randomized, comparative effectiveness phase IV-trial with two treatment arms.

Patients will be randomized 1:1 **either** to valproate **or** levetiracetam treatment arm.

3.2 Requirements at the Trial Sites regarding Personnel and Equipment

The common qualification criteria required by ICH-GCP and the German Medicinal Products Act will be assessed by the ethics committees involved before start of the trial.

3.2.1 Qualification of investigator/deputy and medical staff in the study team

The **coordinating investigator** in multicenter trials and their deputy are licenced to practice medicine, are medical specialists in neurology and have at least two years work experience in patients with status epilepticus. They have theoretical and practical experience in conducting clinical trials. Their qualification is defined as follows:

- Documented proof of at least two years experience in conducting clinical trials after August 2004 (12. Amendment of German Medicinal Products Act) incl. proof of GCP training

AND

- Updates of GCP knowledge and revisions of German Medicinal Products Act every two to three years, if necessary

Investigator and deputy in the participating trial sites are licenced to practice medicine, have completed at least two years of specialist medical training in neurology, including one year in intensive care medicine and have at least two years work experience in patients with SE.

They have theoretical and practical experience in conducting clinical trials. Their qualification is defined as follows

- Documented proof of the conduct of several clinical trials after August 2004 (12. Amendment of German Medicinal Products Act) incl. proof of GCP training

AND

- Updates of GCP knowledge and revisions of German Medicinal Products Act every two to three years, if necessary

The Investigator is responsible for selecting and assembling the study team members (especially the medical staff) according to the requirements of this trial protocol. Furthermore, the investigator is responsible for training and supervision of the study team and providing all necessary information. This has to be documented.

Medical staff with so-called *extensive delegation* is licenced to practice medicine, has completed at least one year of specialist medical training and has at least one year work experience in patients with SE.

Medical staff with so-called *restricted delegation* is licenced to practice medicine.

All medical staff has at least theoretical experience in conducting clinical trials. The qualification is defined as follows

- Certification of successful participation in an investigator course incl. GCP training

OR

- Documented proof of conducting clinical trials after August 2004 (12. Amendment of German Medicinal Products Act) incl. proof of GCP training

AND

- Updates of GCP knowledge and revisions of German Medicinal Products Act every two to three years, if necessary

The staff at the trial sites will be trained for the eCRF data entry, query management and trial-specific procedures including EEG data transfer to the EEG Reference Board.

3.2.2 Essential technical equipment at the trial sites and involvement of other facilities in the trial

The following equipment is required:

⇛ Trained personnel for sampling, preparation and shipment of blood specimen for analysis at local laboratory (including determination of the drug levels VPA and LEV).

⇒ Refrigerators for short-term storage of lab specimen,

⇒ PC for electronic data entry (electronic CRF),

⇒ PC and internet access to transfer the EEG data,

⇒ Facilities to conduct an EEG for the duration of one hour.

3.3 Trial Sites and Number of Trial Subjects

The trial is planned to be conducted in about twenty-five trial sites in Germany.

The aim is to include a total number of 454 patients evaluable for the primary analysis. Assuming a drop-out rate of about 5 %, a total of 477 patients are to be randomized (see 8.4).

3.4 Expected Duration of Trial

- **Duration per patient:**
  Duration of intervention: 10 minutes, followed by observation until 60 minutes
  Duration of the trial: until discharge from hospital, maximum 30 days after enrollment
- **Duration of the trial:**
  First patient in to last patient out (months): 48
  Recruitment period (months): 47
  Duration of the entire trial, including preparation and analysis (months): 60

The trial formally starts with the randomisation of the first patient (FPI = first patient in), and the formal end of the study is the last visit of the last patient included (LPO = last patient out).

3.5 Premature Termination of the Trial

Premature termination of the trial for a single patient is described in chapter 6.4.

3.5.1 Termination of the Trial at a Single Site

The trial can be aborted at a single site if

- the protocol is not adhered to,
- the quality of data is deficient,
- there is inadequate recruitment.

The coordinating investigator decides whether or not to exclude the site, together with the sponsor and biometrician and the data monitoring committee (DMC) if appropriate.

Investigators and sites no longer participating in the trial must inform the coordinating investigator immediately and should provide justification for the decision. Further treatment of patients still involved in the study is to be arranged together with the coordinating investigator.

CAUTION: Trial site may be temporarily placed on hold or closed by the sponsor if no or insufficiently qualified personnel (investigator/deputy) is available.

3.5.2 Termination of the Whole Trial or of Individual Arms of the Trial

The whole trial will be terminated prematurely if, in the opinion of the coordinating investigator or based on the DMC's recommendations, an unfavourable risk-benefit ratio develops or if continuation of the study no longer appears to be reasonably justified.

The trial can be terminated prematurely by the coordinating investigator in the event of

- serious adverse events / unacceptable toxicity
- changes in the risk-benefit considerations, e.g. as a result of unexpected adverse events
- new insights from other trials
- an insufficient recruitment rate.

The final decision regarding the premature termination of the trial will be made by the coordinating investigator.

Since the trial is subject to German Medicinal Products Act (Arzneimittelgesetz – AMG), the approval can be rescinded or the study can be terminated by the responsible federal authority (Bundesinstitut für Arzneimittel und Medizinprodukte – BfArM) or the responsible ethics committee, too.

In case, the study has to be terminated early (due to scientific, organisational, financial, or other reasons) the coordinating investigator will decide on probably meaningful data analyses after consultation of the responsible biometrician.

Neither a formal statistical analysis plan nor a comprehensive statistical report is mandantory in the situation of early termination although all efforts will be made to publish the meaningful results of trial.

4 TRIAL SUBJECTS

As status epilepticus is a neurological emergency patients are admitted to the hospital or are already hospitalized.

4.1 Inclusion Criteria

Patients must meet ALL of the following criteria:

1. Diagnosis of established status epilepticus defined as:
    - ongoing convulsive seizures (generalized/focal with impairment of consciousness/ focal without impairment of consciousness) for the duration of ≥ 5 minutes

    or

    - 2 or more repetitive convulsive seizures without full recovery of consciousness between the seizures for the duration of ≥ 5 minutes

    or

    - ongoing EEG patterns consistent with definite or possible NCSE according to the Salzburg criteria (Leitinger et al. 2016):

EEG patterns have to be continuously present for at least 10 seconds and the whole EEG recording should be abnormal AND

- epileptiform discharges (spikes, polyspikes, sharp-waves, sharp-and-slow-wave complexes) >2.5 Hz
  or
- epileptiform discharges ≤ 2.5 Hz **with**
  - fluctuation[1] **or**
  - typical spatiotemporal evolution **or**
  - subtle clinical ictal phenomenon

  or
- rhythmic delta/theta activity (>0.5 Hz)

([1]) definition of fluctuation (Hirsch et al. 2012): >3 changes (≤ 1 minute apart) in frequency (by at least 0.5 Hz), >3 changes in morphology, or >3 changes in location (by at least 1 standard interelectrode distance)

or

- clinically defined NCSE (*)

that do not respond to the treatment with an adequate dosage of benzodiazepines. Adequate dosages are AT LEAST:

- Lorazepam 2 mg i.v.
- Midazolam 5 mg i.v./intranasal/buccal/i.m.
- Diazepam 5 mg i.v./rectal
- Clonazepam 1 mg i.v.

The dosage may exceed the minimum adequate dosage as indicated above. The antiepileptic drugs may be administered in fractionated doses.

The last administration of benzodiazepines was 5 to 30 minutes prior to start of intervention.

(*) Clinically defined NCSE may be diagnosed in the following circumstances:

patients with

confusion/ fluctuating mental state **and** (minimal) rhythmic motor activity (as twitching of the arms, legs, trunk or facial muscles, blinking, tonic eye deviation or nystagmoid eye jerking) **or**

comatose/stuporous state **and** (minimal) rhythmic motor activity (see above)

Clinically defined diagnosis of NCSE is only permitted if EEG is not available or justified for verification of NCSE. In particular, clinical NCSE must not be diagnosed if NSCE is suspected, but EEG is ambiguous.

2. Adult patients ≥ 65 years old

3. Written informed consent by patient. Due to the nature of the disease, informed consent cannot be obtained at the time of inclusion in the trial (see chapter 4.3).

4.2 Exclusion Criteria

Patients will be excluded for ANY ONE of the following reasons.

1. Treatment of SE with other antiepileptic drugs/sedatives before enrollment
2. **Intravenous** application of VPA or LEV in the last 24 hours before enrollment.

3. Known or suspected severe liver or pancreatic disease (alcohol addiction, known liver cirrhosis or familial liver diseases, clinical signs of severe liver disease such as ascites, jaundice)
4. Known concomitant treatment with one or several of the following medications: phenobarbital, phenytoin, carbamazepine, carbapenem antibiotics, rifampicin, erythromycin, cimetidine, primidone, mefloquine, fluoxetine, felbamat, lopinavir, ritonavir
5. Known coagulopathy (anticoagulants allowed)
6. Known porphyria, mitochondriopathy and urea cycle disorders
7. Known severe kidney disease (GFR < 30ml/min)
8. Known insulin dependent diabetes mellitus
9. Hypoglycemia (< 3.3 mmol/l)
10. Estimated weight < 45kg.
11. Need for acute neurosurgical treatment.
12. Known cardiopulmonary resuscitation within the last 7 days before enrollment
13. Known hypersensitivity against VPA or LEV
14. Known participation in another interventional trial or known participation in any other interventional trial within one month before enrolment into this trial
15. Known former participation in this trial

4.3 Justification for the Inclusion of vulnerable Populations

As outlined above, SE is a potentially life-threatening condition with overall mortality rates of 20% (Trinka and Kälviäinen 2017). As the outcome of SE worsens with the duration of the condition, patients with SE, in particular those with generalized convulsive SE and focal convulsive SE with impairment of consciousness require immediate medical treatment. In case of nonconvulsive SE there is growing evidence that it results in high morbidity and mortality (DeAssis et al. 2012) so treatment should also not be delayed and start as soon as possible. Due to the nature of the disorder and due to the requirement, as per definition, of failed prior therapy with benzodiazepines, patients with established SE are unable to provide informed consent before the initiation of the trial therapy, neither verbally nor in writing.

Considering the present scientific evidence and clinical practice, the trial offers the so far best available treatment of established SE in the elderly; it also reflects recommendations of international guidelines and takes into account age-related biological characteristics. Drug treatment within this trial is almost certain to be substantially safer as the recommended therapy, which places phenytoin first, regardless of age, and hence exposes elderly patients to considerable risks. Furthermore, as there are insufficient data to justify the choice of one treatment over the other, any of the two drugs, LEV and VAL, can be considered as the best.

Patients included in this trial will benefit from the fact that treating physicians will be educated about recognising SE and its medical treatment.

The following describes the general procedure for obtaining informed consent in the special patient population of this trial; the SE related specifics will be listed and explained for each point:

1) Patients with **generalized convulsive SE or focal convulsive SE with impairment of consciousness** suffer from a life-threatening condition and must be treated immediately. In order to avoid an inacceptable delay of treatment the patient will be included in the trial according to § 41 (1) AMG. The treating physician decides about the inclusion while regarding inclusion and exclusion criteria.
The involvement of an independent medical consultant **can`t be considered** as this would cause an unethical delay in therapy in this life-threatening condition. This procedure

corresponds to the "Gießener Lösung" and is recommended by the *Arbeitskreis Medizinischer Ethik-Kommissionen in der Bundesrepublik Deutschland*, too.

Note! If a living will with a DNR-Order (Do Not Resuscitate) exists and is known at timepoint of inclusion, then the patient is not to be included into the trial.

2) In patients with **focal convulsive SE without impairment of consciousness and patients with nonconvulsive SE** treatment should be started as quickly as possible. Due to the SE and/or the prior therapy with benzodiazepines these patients are not capable of providing an informed consent, either.
Therefore, **the legal or authorized representative (2a)** or an **independent medical consultant (2b)** has to be involved in the process (procedures see below):

2a) Inclusion by a legal or an authorized representative can only be implemented if the process is feasible without an inacceptable delay of treatment. As soon as it becomes clear that it is not possible to inform a legal or an authorized representative in a timely manner, the patient must be included via the independent medical consultant (see 2b).

If a legal or an authorized representative is in charge of the patient and the authorization also covers medical treatment, this person must be informed (personally present, not by telephone) immediately and asked about the possibility of participation of the patient in the trial. The legal or authorized representative then decides whether or not the patient will participate in the trial and **signed the respective ICF** prior to trial treatment.

2b) If a legal or authorized representative cannot be reached immediately the following process should be followed: An independent medical consultant examines the patient and confirms the patient's inability to provide consent as well as the urgency of participating in the trial with possible benefit to the patient. Note that the consultant is not permitted to be involved in the trial. The consultant must provide his decision regarding the justification in written form. Afterwards, the patient will be treated with the trial therapy. The described procedure is also integrated in the "Gießener Lösung" (see 1)).

The following link describes the detailed procedures and documents that must be used to legally implement and document the process of patient inclusion: https://www.uni-giessen.de/fbz/fb11/dekanat/ethikkommission/nichteinwfpers

The documents required for the "Gießener Lösung" and a working instruction that describes different processes concerning the inclusion of the patient are available in the investigator's site file.

If it becomes known after the start of trial treatment that a legal or authorized care relationship exists, the persons concerned must be informed about the study and subsequently decide on the patient's participation. If the legal or authorized representative refuses the patient`s participation in the trial, the necessity of the data already collected will be evaluated according to the regulatory requirements of §40 (3) AMG.

Patients initially unable to provide consent must be informed about the clinical trial as soon as they are able to do so and will then be asked to provide their written informed consent. This must also be documented in the patient file.

Empirical evidence suggests that a large proportion of the patients treated in the trial will regain the ability to give their consent within a short period of time (1 to 3 days after randomisation). Therefore, the establishment of a legal care relationship should only begin when it is foreseeable that the patients will not regain their ability to consent in the near future. If the patient has not regained consciousness 72 hours after inclusion into the trial, the establishment of a legal care relationship should be initiated at the responsible local court. All respective

activities have to be documented in the patient’s medical file.

Subsequently, informed written consent must be obtained from the legal representative.

If a legal or authorized representative has given the informed consent and later on the patient refused to participate in the trial, the necessity of the data already collected will be evaluated according to the regulatory requirements of §40 (3) AMG.

PLEASE NOTE:

In case of confirmation by the independent medical consultant or in emergency situation (AMG §41 (1)) a deferred informed consent from patient, legal or authorized representative has to be given as soon as possible.

If the medical consultant confirms justification of trial participation and the patient or the legal representative thereafter does not give the informed consent, all stored blood samples will be destroyed, but the stored data may continue to be used as described above.

4.4 Participation in more than one Clinical Trial

During the verification of the inclusion and exclusion criteria the investigator/his deputy or authorised medical staff of the trial team checks if the patient is currently participating in any other interventional clinical trial(s). Should this be the case, the patient will not be included.

As far as is known, the legal or authorized representative confirms with his/her signature on the consent form that the patient will not simultaneously participate in another interventional clinical trial.

4.5 Statement on the Inclusion of Dependent Individuals

Due to the emergency situation or the very tight time frame for the treatment of patients during the clinical trial, dependence of the patient on the investigator cannot be ruled out. Not to treat the patient in the study due to a possible dependence on the investigator and to deny him/her a possibly beneficial therapy does not appear to be ethically justifiable. In case of doubt, the patient should not be included in the study by the physician with whom a dependency relationship exists, but by a colleague from the study team, provided that this is feasible in the short time available.

4.6 Rationale for Gender Distribution

With regard to one German investigation about epidemiological issues (Knake et al. 2001) a gender ratio of 1:2 (female:male) can be expected, but no sufficient data exist in the older age group.

5 INVESTIGATIONAL PRODUCT

5.1 Trial Drugs

Generic Name:	Valproic acid (VPA)
Manufacturer:	Department Pharmacy of the University Hospital Erlangen
Storage conditions:	≤ 30 °C

Stability:	6 months (Re-Test Date)
Generic Name:	Levetiracetam (LEV)
Manufacturer:	Department Pharmacy of the University Hospital Erlangen
Storage conditions:	≤ 30 °C
Stability:	6 months (Re-Test Date)

The *Investigator Medicinal Product Dossiers* (IMPD) contain detailed information on the quality, manufacture and analysis of the investigational products Valproic acid and Levetiracetam. For clinical information, reference documents are the Summary of medicinal Product Characteristics (Fachinformationen, see 5.5.2*)* and instructions for use *(Gebrauchsanweisung)* for Valproic acid and Levetiracetam.

All documents mentioned are part of the submission documents.

5.2 Packaging and Labelling of the Trial Drug

The investigational products will be manufactured, packaged and labelled by Department Pharmacy of the University Hospital Erlangen according to the current standards of Good Manufacturing Practice (GMP).

The trial drug will be labelled as required by the ICH-GCP Guideline E6 and the German Medicinal Products Act (AMG § 10). The labelling will be in the official language of the country in which the trial takes place.

The master label is part of the documents that will be submitted to the responsible federal authority.

One blinded patient-specific trial drug (VAL **or** LEV) with identification number is kept in a separate package, which also contains the unblinding envelope and instructions for use.

The trial medication will not be shipped before all requirements for the start of the clinical trial have been fulfilled according to the internal SOP.

5.3 Storage and Handling Requirements

The investigator shall take responsibility for and shall take all steps to maintain appropriate records and ensure appropriate supply, storage, handling, distribution and usage of investigational product in accordance with the protocol and any applicable laws and regulations.

Clinical supplies must be stored in a secure, limited-access location under the storage conditions specified above.

Receipt and dispensing of trial medication must be recorded by an authorised person at the trial site.

Clinical supplies may not be used for any purpose other than that stated in the protocol.

5.4 Drug Accountability

After initiation of trial site by the sponsor (executed by the ZKS Leipzig) the delivery of study medication is released. Blinded trial drugs (VAL **or** LEV) will be provided to the trial sites by

Department Pharmacy of the University Hospital Erlangen. The trial drug may only be used within the framework of this clinical trial and in accordance with this trial protocol.

The scope of drug delivery (packages) depends e.g. on the shelf life of the trial medication as well as on recruitment in the further course of the trial.

All shipments will be coordinated by ZKS Leipzig.

Upon receipt of study medication the trial site will fax the medication receipt form to the Department Pharmacy of the University Hospital Erlangen. Trial sites will complete and update a site inventory log about all medication received at the site.

Storage of study medication will strictly follow of the manufacturer’s storage recommendation. Temperature logs recorded from min-max thermometers will document the appropriate storage conditions for the respective study medication.

The medical staff at the trial site and the monitor from the ZKS Leipzig evaluate the drug accountability as part of the on-site checks.

For each study subject an individualized drug accountability log will be recorded at trial site.

In the course of the trial unused trial medication and empty packages are collected and destroyed at trial site.

The application of trial drugs is documented in the patient’s medical record AND the respective parts of the Case Report Form (CRF). Orderly disposal of trial drugs will be accomplished and documented by the investigator/designated personnel. Storage in the trial sites and administration to patients will be documented by the investigator/designated personnel.

Upon completion or termination of the trial, all unused and/or partially used investigational product will be destroyed at the site per institutional policy provided that procedures for proper disposal have been established according to applicable federal, state, local and institutional guidelines. The investigator is responsible to arrange for disposal of all empty trial drugs and to keep appropriate records of disposal.

The site monitor will assure that a final report of drug accountability is prepared and placed in both the Investigator Site File (ISF) and the Trial Master File (TMF).

5.5 Administration of the Study Drugs

5.5.1 Procedures

The trial drugs (**either** LEV **or** VPA) shall be administered in a single infusion over **10 minutes**. Therefore, a peripheral intravenous access has to be established according to the clinical standards. The provided formulation includes the maximum possible dosage of 4.5 g LEV **or** 3 g VPA. Always the whole fluid (50 ml) shall be transferred to the syringe for direct use. The perfusor running rate depends on the (estimated) weight.

To patients with a weight of 100 kg or higher the whole 50 ml will be infused, in the other cases the remaining fluid will be discarded.

The start of infusion has to be realized as soon as possible. The infusion has to be stopped in preterm if:

- there are clinical signs of an allergic reaction
- the investigator decides to do so regarding safety issues

In case of cessation of ictal activity before the end of infusion the application has to be finished anyway. The infusion can only be stopped preterm if the investigator rates a risk that justifies this decision.

The trial drugs will be administered as following:

The dosages for the patients are:

- LEV 45 mg/kg, VPA 30 mg/kg

The perfusor running rate will be adjusted in the following manner:

Estimated body weight	Running rate (ml/h)
45-49 kg	135
50-54 kg	150
55-59 kg	165
60-64 kg	180
65-69 kg	195
70-74 kg	210
75-79 kg	225
80-84 kg	240
85-89 kg	255
90-94 kg	270
95-99 kg	285
100 kg and more	300

5.5.2 Dealing with Side-effects

To ensure the appropriate recognition and treatment of complications during the intervention this chapter focusses on possible side-effects of the **i.v.application** of VPA or LEV.

In safety studies the following side-effects of **VPA** have been reported:

- Transient: dizziness, headache, somnolence, taste perversion, nausea, vomiting, injection-site reactions (Devinsky et al. 1995)
- Transient hypotension (Ramsay et al. 2003)
- Asymptomatic hyperammonaemia (DeWolfe et al. 2009)
- Respiratory depression (Trinka et al. 2014)
- Hepatic abnormalities (Trinka et al. 2014)

Case reports also described encephalopathy with or without concomitant hyperammonaemia, acute hepatic failure and acute pancreatitis (reviewed in Trinka et al. 2014).

According to the review (Trinka et al. 2014) about experiences with cumulative 860 patients suffering from various types of SE and treated with i.v. VPA dizziness, thrombocytopenia and mild hypotension are the reported most common side-effects. With regard to the prescribing information (Fachinformation Orfiril® Desitin) the following possible side-effects should also be considered:

- metabolic acidosis, renal failure (frequency unknown)
- Leukopenia, elevation of liver enzymes (often, 1 to 10 of 100 treated subjects)
- Tremor, confusional state (often)
- Bleedings, severe hepatic damage (occasional, 1 to 10 of 1000 treated subjects)
- Severe pancreatic damage (rarely, 1 to 10 of 10.000 treated subjects)

In retrospective case series and safety studies on over 1000 patients including more than 500 patients with SE so far **LEV** has most often been associated with somnolence and sedation (Trinka et al. 2015). In single cases LEV has been associated with delirium (Hwang et al. 2014; Kumar et al. 2014; Foley and Bugg 2010).

Also the following possible side-effects have been reported (Fachinformation Levetiracetam UCB®):

- nasopharyngitis, headache, dizziness (very often, 1 or more of 10 treated subjects)
- agitation, anxiety, hostility, insomnia, depression (often, 1 to 10 of 100 treated subjects)
- nausea and vomiting (often)
- suicidal ideation (occasional)
- thrombocytopenia, leukopenia (occasional)
- cognitive impairment (occasional)
- pancreatitis, severe hepatic or renal damage, encephalopathy (rarely)

The ESET trial (Kapur et al 2019) reports a depressed level of consciousness and respiratory distress as most frequent serious adverse events. The incidence of these events did not differ significantly between the treatment groups (LEV, VPA and fosphenytoin). Numerically depression of consciousness (15 vs. 9) and respiratory depression (10 vs. 8) was more often in the LEV treatment arm than in the VPA treatment arm (Supplement to Kapur et al. 2019).

During the intervention of ToSEE the relevant acute side-effects as hypotension and respiratory depression are recorded by the cardiopulmonary monitoring. In addition at least one member of the trial staff will guard the patient and check his clinical status repeatedly during 60 minutes after the start of the intervention. According to the clinical standard of SE-treatment rescue medication and airway-management shall be provided. The lab analysis before the intervention and after the observation period serves the detection of pre-existent and acute hepatic or renal impairment.

5.5.3 Counterindicated/Forbidden Concomitant Medication

According to the potential influence on the plasma level of VPA the following drugs should be avoided during 60 minutes after start of infusion if the responsible investigator approves it justifiable:

- Anticonvulsant drugs: phenobarbital, phenytoin, carbamazepine, felbamat
- Anti-infectives: carbapenem antibiotics, rifampicin, erythromycin, mefloquine, lopinavir, ritonavir
- others: cimetidine, primidone, fluoxetine.

A previous treatment with the anti-infectives mentioned above is **NOT** an exclusion criterion.

5.5.4 Overdose and Abuse

None of the two study drugs has known abuse potential. In addition, only one single infusion will be administered. The tolerability of both drugs and of the application regime in elderly and old patients is to investigate. To serve this goal clinical data including adverse events together with the comparison of plasma drug levels before and 60 minutes after the start of infusion will be considered.

5.6 Blinding and Unblinding

The trial is a double-blind trial. The trial medication (VAL **or** LEV) will be manufactured by Department Pharmacy of the University Hospital Erlangen (see chapter 5.1). Blinding will also be conducted by the manufacturer.

The randomisation list will be produced by the responsible trial biometrician at the ZKS Leipzig. The original list will contain the allocation of the treatment arm to a corresponding identification number of a study drug package (details to the randomisation process see 8.1.1).

Randomisation will be blinded to the patients and the study site staff. The drugs are both formulated in transparent liquids of the same appearance. Also the running rates of infusion are identical so the blinding can be sustained over the time of intervention.

The unblinding will be implemented by envelopes (sealed and light-proof) provided in the packages. The responsible investigator has to state the time point and reason for unblinding. Also he/she has to confirm in a written form that the unblinding was executed at a time after the end of 15 minutes after the start of the intervention **or** to justify the deviant procedure.

6 INDIVIDUAL TRIAL PROCEDURES

6.1 Patient Information and Informed Consent

Regarding the addressed disease patients will not be able to provide informed consent at the time of inclusion. As a result, patients are always informed only **after** the study intervention.

The details of the procedures for patients who are not able to provide informed consent in person are described in section 4.3.

In the following, the general procedure for receiving an informed consent is explained. In the case of the ToSEE trial, this will be done if the patient regains the ability to consent or if a legal or an authorised representative is consulted.

In accordance with international guidelines, the informed consent of trial participants will be in writing (written, dated and signed by the person performing the interview referred to below, and by the subject).

The patient’s consent must refer explicitly to the collection and processing of health-related data. Therefore the patient, a legal or an authorised representative should be informed explicitly about the purpose of collecting the data and scope of what is to be collected and that personal data, including health related data, will be stored and used for analyses in a pseudonymized form.

Before obtaining informed consent, the potential trial participant, a legal or an authorised representative will receive information regarding the clinical trial in an interview. It will be performed by a qualified medical member of the trial group authorised by the investigator for this task.

The interview has to cover the following items:

- the nature, objectives, benefits, implications, risks and potential inconveniences of the clinical trial
- the expected duration of the subject's participation in the clinical trial
- the information that the patient, a legal or an authorised representative may withdraw his/her consent to participate at any time without giving reasons. The patient is to be informed that in case of revocation of his/her consent, the stored data may be used

further, as may be necessary to

  - o assess effects of the medicinal product being tested,
  - o guarantee that the patient's personal interests are not adversely affected,
  - o comply with the requirement to provide complete authorisation documentation.

- potential treatment alternatives
- follow-up measures in case of early termination of the trial for the patient or overall
- the applicable damage compensation system in case of damage to a patient
- the right on data access, rectification and withdrawal of personal data

The trial subject, a legal or an authorised representative will have the opportunity to ask questions at any moment.

The signed original form has to be retained at the trial site, one copy or a second signed exemplar shall be handed out to the patient or the legal or authorized representative.

6.1.1 Withdrawal of Informed Consent

Patient (**or** legal or authorised representative) may withdraw their consent to participate at any time without giving reasons. Nevertheless, the patient should be asked for the reason of the premature termination after being informed that he/she does not need to do so. Information as to when and why a patient was registered/ randomized and when he/she withdrew consent must be retained in the documentation.

The patient is to be informed that in case of revocation of his/her consent, the stored data may be used further (according to § 40 lit 2a sublit 3 to 5 German Medicinal Products Act), as may be necessary to

- assess effects of the drug being tested,
- guarantee that the patient's personal interests are not affected adversely,
- comply with the requirement to provide complete authorisation documentation.

Data no longer required for the aforementioned purposes shall be deleted immediately.

6.2 Enrolment in the Trial

The inclusion of patients with convulsive SE follows the consideration of the inclusion and exclusion criteria without further diagnostic procedures. The diagnosis NCSE usually requires the confirmation by EEG.

Generally, the local investigator at the trial site may **pre**screen subjects for general participation in the current study on the basis of pre-existing data (diagnosis status epilepticus and age ≥ 65 years). All prescreened patients are listed on a pre-screening list.

NOTE: Procedures performed solely for the clinical study can be performed only after inclusion procedures described in chapters 4.3 and 6.1.

At first, general inclusion/exclusion criteria are checked, demographic data are recorded, blood sampling for local laboratory test and if any, further examinations are performed in order to check all inclusion and exclusion criteria - before randomisation (see 6.2.1*).*

6.2.1 Screening

<u>The screening assessments are:</u>

- Assessment of SE status/type using standardized methods
- Note the probable/definite start of SE
- Note the time of the **last** application of benzodiazepines before the intervention
- Assessment of concomitant diseases and medication, as detailed as appropriate
- <u>Further clinical assessment</u>
    - Assessment of vital signs (arterial blood pressure, heart rate, temperature, respiratory rate, estimated body weight) according to clinical standard

- Local lab analysis (EDTA and heparine-plasma and/or serum)
    - <u>Liver function test including</u> alanine aminotransferase, aspartate aminotransferase, gamma-glutamyl-transferase
    - <u>Kidney function test including</u> creatinine, blood urea, glomerular filtration rate,
    - level of valproate, level of levetiracetam,
    - blood count,
    - sodium,
    - blood glucose (if not yet evaluated)

Blood sampling for local lab analysis is performed in parallel with the preparation of the study drug. If the blood sampling could not be completed before the start of the infusion, the procedure must be initiated simultaneously. The time of blood sampling is noted.

Information derived from blood samples taken as part of the SE-treatment in the clinical routine can also be used.

Note!

Blood sampling and subsequent local lab analysis must not delay start of the patient's treatment.

- Assessment of the GCS,
- perfom brief neurological exam: orientation, horizontal extraocular movements, motor drift of both arms and legs, sensation, language
- Obtain the mRS, BI and Home care before admission – if it possible by relatives (see **During 24 hours after T0**)

6.2.2 Randomisation

As soon as all assessments required for the final check of eligibility are available, the patient will be randomized directly by the trial site as following:

The allocation of treatment will be done with the help of the packages. The medical staff in the trial sites is compelled to take the set with the lowest ID number ever (see chapter 8.1.1). The identification number of the trial drug package has to be linked to a patient identification number provided on the patient identification list (PIL). Within 24 hours (working day) after the randomisation the patient is registered in the database by the trial site. Following, the eCRF will be provided.

6.2.3 Discovery of a Violation of the Eligibility Criteria after the Fact

In general, the violation of eligibility criteria is not a reason for premature withdrawal of the patient from the trial therapy or from the whole trial.

If it is discovered that the patient was not eligible at the time of enrollment, this has to be reported to the Data Management (ZKS Leipzig) as soon as possible. After discussing the best decision for the individual patient and his/her inclusion in the full analysis population between the investigator and the biometrician (e.g. via phone) the ZKS Leipzig informs the investigator /his deputy or authorised medical staff immediately as to what is to be done with the patient. The patient's data will further be recorded.

6.2.4 Description and justification for the route of administration, dosage, dosage regimen, and treatment period(s)

As longer duration is associated with higher morbidity and mortality (Madzar et al. 2016) SE has to be terminated as quickly as possible. Furthermore recurrence of ictal acitivity is to avoid. To fulfil the conditions of cessation and stable control one infusion over 10 minutes is planned to generate a high plasma level of an effective drug within a short time. The regimen of one treatment period has also been applied in interventional trials of the past (i.e. Gilad et al. 2008, Treiman et al. 1998, Kapur et al. 2019). The suggested dosages of both trial drugs (VPA 30 mg/kg, LEV 45mg/kg) and the dosage regimen (mg/min) are in accordance to the German guidelines for the treatment of SE in adults (Leitlinien, Deutsche Gesellschaft für Neurologie). In 10 published controlled trials with children and adults of all age groups VPA was administered most often at an initial bolus of 20 or 30mg/kg. Although separate safety studies of the older population are missing the observations of the available studies revealed a good cardiovascular and respiratory tolerability in patients with multiple comorbidities despite high dosages and infusion rates (Sinha and Naritoku 2000, Trinka et al 2014). LEV has shown a favorable tolerability profile in published experiences of over 500 patients who suffered from SE. (Trinka et al 2015) There are no data about SE-treatment in elderly with compromised renal function that would deliver a dosage regimen. In a small retrospective analysis LEV plasma levels measured less than 36 hours after loading and response rate in older people showed no clear benefit of dosages between 20 and 30 mg/kg, so authors suggest a further increasing up to 60mg/kg (Perrenoud et al. 2018). In consideration of the reduced renal elimination on the one hand and the strict demand for efficacy on the other we propose a dosage of 45mg/kg. Frequent controls of renal function and LEV plasma levels beyond the 2 lab controls before and 60 minutes after the intervention are demanded in the clinical setting. With regard to the safety profile of both drugs and the potentially serious or even fatal consequences of SE the ratio of benefit to risk seems clearly in favor of treatment.

6.3 Description of the Treatment Procedures

<u>Visit 1 (V1)</u>

Before initiation of intervention

- Setting-up a cardiopulmonary monitoring capable to measure heart rate, oxygen saturation and blood pressure
- **In case of NCSE**: Stop the clinical routine EEG recording before the initiation of intervention and start a new one labeled by the patient ID and WITHOUT further identification data.
- **There must be no delay in the administration of the study drug. The results of the local lab analysis performed at the time of the screening (exception: blood glucose, if unknown) must not be waited for.**

During 60 minutes after initiation of intervention

- Monitoring of blood pressure as clinically indicated, heart rate and oxygen saturation continuously during 60 minutes after initiation of infusion. Only parameters requiring emergency treatment (as hypotension, tachyarryhtmia, low oxygen saturation) have to be recorded.
- **In case of NCSE**: It is not possible to replace the EEG device AFTER the start of intervention. The EEG has to be recorded until 60 minutes after the start of intervention

T0 - Initiation of intervention

Details to the administration of study drugs – see 0

- Note the time of initiation
- Check for the availability of the unblinding envelope

T15 – 15 minutes after initiation of intervention

- Check for cessation of convulsive seizures or minimal rhythmic motor activity or cessation of EEG signs of NCSE according to Salzburg criteria (Leitinger et al. 2016)
    - Cessation of EEG signs of NCSE defined as:
        - No rhythmic epileptiform discharges that are continuously present for at least 10 seconds
        - No rhythmic delta/theta acitivity **or** rhythmic delta/theta activity ≤ 0.5 Hz that is continuously present for at least 10 seconds
- Administer GCS

T30 – 30 minutes after initiation of intervention (+/- 5 minutes)

- Administer GCS

T60 – 60 minutes after initiation of intervention (+/- 5 minutes)

- Administer the GCS and perfom brief neurological exam: orientation, horizontal extraocular movements, motor drift of both arms and legs, sensation, language
- Obtain a blood sample (EDTA and heparine-plasma and/or serum) for the following parameters:
    - blood count
    - alanine aminotransferase, aspartate aminotransferase, gamma-glutamyl-transferase
    - creatinine, blood urea, glomerular filtration rate
    - sodium
    - level of valproate, level of levetiracetam

During 45 minutes after T15 - until T60

- Check for the recurrence of convulsive seizures or minimal rhythmic motor activity or recurrence of EEG signs of NCSE according to Salzburg criteria
- Unblind the medication in case of recurrence of ictal activity (envelope), the subsequent procedure is to be executed in accreditation of the treating physician (as watch and wait/ repetition of infusion with the same antileptic drug/ further treatment with another antiepileptic drug)
- Note the time of unblinding (how many minutes after T0? – must be ≥ 15 min)

During 24 hours after T0

- Obtain written (signed, dated) informed consent at the time the patient has regained consciousness and is able to provide informed consent
- Obtain the pre-mRS and BI **(retrospective)** and Home care before admission in a survey with the patient or a proxy **– see Screening**
- Patients with NCSE: transfer the EEG data to the cloud (according to the working instructions)

From initiation of intervention (T0) until the end of the study

- Assess for adverse events (chapter 7)

Visit 2 (V2)

Day of discharge/day before discharge from hospital or day 30 (+/- 1 day), whatever is sooner

- Record the following data (eCRF)
    - day of recurrence of seizures or convulsive SE after successful intervention/ day of detection of NCSE (EEG) after successful intervention
    - day of first EEG-detection of terminated NCSE after failed intervention
    - further antiepileptic treatment after intervention: medication, dosage, type and frequency of application including stop of medications
    - reason for withdrawal of treatment with VPA or LEV
    - acute renal failure with or without dialysis
    - acute pancreatitis
    - acute hepatic failure
    - lab analysis: level of ammoniaemia and subsequent repetitions, as ordered by treating physician *
    - type of infections requiring i.v. antibiotic treatment
    - start and stop of invasive/noninvasive ventilation (day)
    - development of delirium and treatment
    - complications requiring surgery or invasive diagnostics/ therapy
    - etiology of SE (cerebral focus, metabolic/infectious/toxic conditions, noncompliance/withdrawal/change of antiepileptic medication)
    - home care after hospital stay (if applicable)
- Administer GCS and perform brief neurological exam: orientation, horizontal extraocular movements, motor drift of both arms and legs, sensation, language.
- Obtain mRS and BI

* **NOTE:** As soon as an otherwise unexplained disturbance of consciousness, nausea or hypotension occurs in duration of treatment with VPA during the hospital stay the efforts of clarification **must** include the detection of a potential hyperammonemia.

6.4 Premature Termination of the Trial

Because the endpoint is assessed until 60 minutes only few patients will terminate the study prematurely: This may be patients who or whose representives withdraw a former informed consent.

The primary statistical analysis follows the intention to treat principle as closely as possible. For a valid analysis, it is of great importance to minimise the rate of drop-outs. Therefore, in patients who do not withdraw their consent, all trial visits shall be performed as scheduled.

In case of premature termination of trial, it is necessary to document the date (as exactly as possible), the reason of termination and the current condition of the patient. Therefore, the eCRF “End of study (ES)” has to be completed for each patient. Data entry to this eCRF page will trigger an automatic report to the responsible trial team members at the ZKS Leipzig.

The “End of study”- eCRF routinely contains the following data:

- Date of individual end of trial
- Reason for trial termination

6.4.1 Premature Termination of the Intervention for Individual Patients

The date and if possible the circumstances and reasons for every premature termination of the therapy will be recorded by the site where the patient was being treated and will be reported to the ZKS-Data Management and the Coordinating Investigator or his deputy.

The intervention has to be stopped for any of the following reasons:

- the patient experiences a hypersensitivity or suspected allergic reaction to study treatment
- the patient experiences a medical emergency that necessitates the discontinuation of treatment
- the patient experiences a medical emergency that necessitates the unblinding of treatment
- patient withdraws his/her consent to participate
- as decision of the investigator

The discontinuation of the intervention and the circumstances must be reported in the eCRF. The study documentation will be continued unless the patient withdraws the consent for participation.

With exception of the rules described above, premature termination of therapy should be avoided.

All further trial visits will take place as planned and described in section **Fehler! Verweisquelle konnte nicht gefunden werden.**.

Termination of trial therapy does not mean that the patient is off-trial.

6.4.2 Premature trial termination for individual patients

All randomised patients will be followed up until Visit 2. Premature termination of trial therapy does not necessarily lead to individual trial termination (see chapter 6.4.1 for explanation).

The only circumstances in which a premature trial termination (i.e. no further trial visits) in a randomised patient is unavoidable are:

- The clinical condition of a patient is worsening (and not responding to treatment) that contraindicates permanently participation in any trial visits

- withdrawal of informed consent or
- death of the patient.

6.5 Plan for Further Treatment

Beyond the single infusion the treatment of SE is determined by the responsible clinicians and follows clinical standards. The possible further treatment with one of the trial drugs VPA or LEV will be observed according to the study design.

7 ADVERSE EVENTS (AE/SAE)

7.1 Adverse Events (AE)

7.1.1 Definition Adverse Event

An Adverse Event (AE) is any untoward medical occurrence in a patient or clinical investigation subject administered a pharmaceutical product and which does not necessarily have to have a causal relationship with this treatment (ICH-Guideline E2A).

Adverse Events encompass any unfavourable and unintended sign (including an abnormal laboratory finding, for example), symptom, or disease that arise newly or worsen after the inclusion of the patient into the trial.

7.1.2 Definition Adverse Reaction

In the pre-approval clinical experience with a new medicinal product or its new usages, particularly as the **therapeutic dose(s) may not be established**: all noxious and unintended responses to a medicinal product related to any dose should be considered adverse drug reactions. The phrase "responses to a medicinal products" means that a causal relationship between a medicinal product and an adverse event is at least a reasonable possibility, i.e., the relationship cannot be ruled out (ICH Guideline E2A).

7.1.3 Documentation and Reporting

Adverse events (AE) will be documented for patients in both treatment arms (VPA and LEV) **from the moment of initiating the infusion of IMPs until the end of the study.**

AE reports comprise any newly reported events as well as worsening of pre-existing conditions (i.e. increased intensity/grading) during the predefined reporting period of events.

AEs are documented on specified AE forms provided in the CRF.

If an AE fulfils any of the criteria for an SAE (see chapter 7.4 for SAE-definition), both the AE pages of the CRF **and** the SAE form must be completed. This applies to all SAEs, **whether or not they are considered to be related to trial treatment.**

For both serious and non-serious AEs, documentation should be supported by an entry in the patient’s heath record. Required information in the patient’s health record should include:

- type of AE
- grade/severity/intensity acc. to e.g. NCI CTCAE 5.0
- seriousness (see also section 7.4)
- onset date

- end date
- actions required
- outcome
- assessment of its relationship to trial drug

All abnormal physical and/or laboratory results which are considered to be clinically relevant by the investigator should be recorded as AEs. If an abnormal laboratory result meets any of the criteria for a SAE, this must also be reported on the SAE Form.

Adverse Events are classified by their seriousness, intensity/severity and relationship to the IMP (see also 16.1).

The following events could be SE-related clinical outcomes/endpoints. Occurrence will be documented as (S)AEs and furthermore within the CRF in addition and independently from the (S)AE-form:

- Indication for the need of an emergency medication (different from allocated study drug) during 60 minutes after initiation of infusion
- Respiratory depression requiring noninvasive/invasive ventilation (oxygen supply excluded) during 60 minutes after initiation of infusion
- SE-recurrence/seizures after successful intervention
- SE- associated ventilation until hospital discharge
- Delirium
- Infections treated with i.v. anti-infectives (type of infection)
- Other complications: sedation, dizziness nausea, vomiting, new thrombocytopenia, leukopenia, new elevation of liver enzymes, acute new liver failure or pancreatic damage, hyperammonaemia, tremor, psychiatric complications.

7.2 Safety Analysis

During the course of the trial, every patient will be monitored closely. Before the initiation of the infusion and during 60 min following initiation of the infusion the following safety assessments will be performed:

- Neurological assessments
- Cardiopulmonary monitoring during the whole time of infusion until 60 minutes after initiaton of infusion
- Laboratory assessments before and 60 minutes after initiation of infusion
- Furthermore safety monitoring encompasses documenting Adverse Events. Adverse Events will be reported until the end of the study.

7.3 Concomitant Diseases

At screening all relevant ongoing or past diseases or medical conditions will be recorded as detailed as possible. Deterioration of a pre-existing condition during the trial will be reported as an adverse event.

7.4 Serious Adverse Events/Serious Adverse Reactions (SAE/SAR)

7.4.1 Definition

An Adverse Event/Reaction is defined to be serious according to ICH-Guideline E2A, paragraph IIB [2] §3 (8) GCP-V [3], if it

- **results in death**,
- **is life-threatening**,

  Note: The term "life-threatening" in the definition of "serious" refers to an event in which the patient was at risk of death at the time of the event; it does not refer to an event which hypothetically might have caused death had it been more severe.

- **requires in-patient hospitalisation or prolongation of existing hospitalisation[1]**,
- **results in persistent or significant disability/incapacity or**
- **is a congenital anormal/birth defect** .

Medical and scientific judgement should be exercised in deciding whether expedited reporting is appropriate in other situations, such as important medical events that may not be immediately life-threatening or result in death or hospitalisation but may jeopardise the patient or may require intervention to prevent one of the other outcomes listed in the definition above. These should also usually be considered serious.

7.4.2 Documentation and Reporting Obligations: INVESTIGATOR

Serious Adverse Events have to be documented on the SAE-forms and the investigator must report them to the sponsor immediately. If more information about the SAE becomes available later, it must also be reported to the sponsor immediately. Serious adverse events (SAE) will be documented for patients in both treatment arms (VPA and LEV) **starting at the moment of initiating the infusion of IMPs. All SAEs** will be documented **until the end of the study** (Visit 2).

The Serious Adverse Events that are also endpoints are to be reported as described in chapter 7.1.3.

In the event of a patient's death, the investigator/ the deputy or the authorised medical staff provide the leading ethics committee(s), all involved ethics committees in multi-centred trials, the responsible federal authority and the sponsor with all further information needed to fulfil their tasks **upon request**.

In all the reports, personal data are to be pseudonymized by using the patient's identification code. It must be possible to relate the initial and all follow-up reports to each other by means of the patient identification number, name and address or the like.

The investigator, the deputy or the authorised medical staff must report every Serious Adverse Event as soon as it is known to the following address:

ZKS Leipzig / Arzneimittelsicherheit
Universität Leipzig

---

[1] In general, hospitalisation means that the patient stays (usually at least an overnight stay) at the hospital or emergency ward for observation and/or treatment. Complications that occur during the stay are AEs. If a complication prolongs hospitalisation or fulfills any other serious criteria, the event is serious.
Hospitalisation for elective treatment of a pre-existing condition that did not worsen from baseline is not considered an AE.

Zentrum für Klinische Studien Leipzig
Härtelstr. 16-18, 04107 Leipzig
Telefon: +49/341/97-16129
E-mail: pharmacovigilance@zks.uni-leipzig.de

Fax: +49/341/97-16278

7.4.3 Documentation and Reporting Obligations: SPONSOR

After the ZKS Leipzig receives the SAE, it is immediately passed on to the coordinating investigator/responsible person for medical assessment.

The coordinating investigator/responsible person forms a second medical opinion of the SAE with respect to causal relationships and the decision as to whether or not it was expected, as described in chapter 16.1.3 and 16.1.4 and forwards the second opinion to the ZKS Leipzig within two days of its arrival.

In the ZKS Leipzig the SAE data are entered into the SAE database immediately and the MedDRA coding takes place simultaneously.

Then forwarding as per law and as described in Chapter 7.6 only for Suspected Unexpected Serious Adverse Drug Reactions (SUSARs) takes place.

Details of the sponsor's documentation and reporting obligations will be specified in a special, trial-specific pharmacovigilance plan, which will be written and finalised alongside with this protocol, if possible.

7.5 Periodic Reports

7.5.1 Annual Safety Report

According to German Medicinal Products Act the sponsor writes a safety report annually (Annual Safety Report, ASR[2]) and sends this report to the leading ethics committee and the federal authority.

Should there be additional regular reports (Periodic Safety Reports – PSR) required by ethics committee and/or authority according to the approval statement, these will also be provided by the sponsor.

The key date is the date of the first authorization of the clinical trial by a federal authority. All data obtained up to this date (each year) will be included in the ASR. Beginning with the key date, there is a time-limit of 60 days for the preparation and submission of the ASR.

The ASR will be prepared by the ZKS Leipzig (project manager, responsible biometrician and PV data manager) acc. to the national requirements Deadlines regarding the ASR are based on the German Medicinal Products Act.

The final report will be released in co-operation with the principal investigator/sponsor's representative.

[2] See "Detailed guidance on the collection, verification and presentation of adverse reaction reports arising from clinical trials on medicinal products for human use").

7.6 Suspected Unexpected Serious Adverse Reactions (SUSAR)

7.6.1 Definition

Suspected Unexpected Serious Adverse Drug Reactions (SUSARs) are side-effects (there is at least a reasonable possibility for a relationship between the Adverse Event and the administration of the investigational product, i.e., the relationship cannot be ruled out), the nature or severity of which are inconsistent with the information available about the product. Information about the trial products are contained in the SmPCs (Summary of medicinal Product Characteristics*)*.

7.6.2 Documentation und Reporting Obligations

Information *for SPONSOR*

The sponsor submits all information available about a SUSAR immediately to the leading ethics committee, the responsible federal authority, and to all participating primary investigators, at the latest within 15 calendar days after the event becomes known.

For every SUSAR that results in death or a life-threatening condition, the leading ethics committee, the federal authority and all participating investigators must be informed by the sponsor within 7 calendar days after the event becomes known. Additional information has to be given within 8 further calendar days.

Details of the sponsor's documentation and reporting obligations will be specified in a special, trial-specific pharmacovigilance plan which will be written and finalised alongside with this protocol, if possible.

Information for INVESTIGATOR

The investigator passes down all relevant information concerning the SUSAR to all participating trial investigators at his/her trial centre. This has to be confirmed by the investigator by signing an acknowledgement document.

To ensure that the investigators and all other trial personell remain "blind" in the case of a SUSAR, the investigating centres will receive all potential SUSARs without information concerning the administered drug (i.e. regardless of whether the patient was treated with LEV or VPA).

7.7 Other Safety Relevant Issues

Other safety issues also qualify for expedited reporting where they might materially alter the current benefit-risk assessment of an investigational medicinal product or would be sufficient to consider changes in the investigational medicinal products administration or in the overall conduct of the trial, for instance:

New events related to the conduct of a trial or the development of an IMP likely to affect the safety of subjects, such as:

- a serious adverse event which could be associated with the trial procedures and which could modify the conduct of the trial,
- a significant hazard to the subject population such as lack of efficacy of an IMP used for the treatment of a life-threatening disease,
- a major safety finding from a newly completed animal study (such as carcinogenicity),
- a temporary halt of a trial for safety reasons if the trial is conducted with the same investigational medicinal products in another country by the same sponsor
- Recommendations of the DMC, if any, where relevant for the safety of subjects.

The sponsor, together with the DMC if appropriate, decides if the number of events or qualitative changes in the expected SARs comprise a safety issue and must be reported.

7.8 Therapeutic Procedures.

If a patient requires treatment as a result of an Adverse Event, then it must meet the recognized standards of medical care in order to restore the patient’s health. Appropriate resuscitation devices and medication must be available in order to treat the patient as quickly as possible in the event of an emergency.

The action taken to treat the AE/SAE must be documented by the investigator either in the appropriate CRF and/or using additional documents.

7.9 Dealing with Pregnancy

Regarding the planned study population pregnancys are not expected events in the course of the trial.

8 BIOMETRY

8.1 Biometrical Aspects of the Trial Design

8.1.1 Measures to Prevent Bias

Randomisation

Randomisation in a 1:1 ratio is performed as block randomisation with randomly varying block length stratified by centre.

Randomisation procedure

Randomisation lists are created by statistical software. Blocks of randomly chosen length are created as realisations of random samples and stringed together to a list.

ZKS Leipzig delivers one randomisation list per centre each to the central pharmacy Erlangen. Beginning with the first entry in the randomisation list, the staff of the pharmacy packs medication kits for one patient each. Every kit is labelled by a consecutive ID number and contains a sealed opaque envelope revealing the information about the medication. A pre-defined number of kits are packed into a package and are sent to the trial sites. The medical personal in the trial sites is obliged to take the set with the lowest ID number. Treatment failure is defined as clinical evidence of epileptic activitiy and / or persistent electroencephalographical ictal activity consistent with NCSE according to the Salzburg criteria 15 minutes after the initiation of intervention or recurrence of ictal activity (in case of NCSE return of EEG patterns to baseline status after initial treatment response) during the further 45 minutes observing period which leads to immediate unblinding of medication. In case of treatment success, drug therapy will be unblinded after 60 minutes to ensure appropriate further treatment (see Figure 1). The investigator is responsible that the envelope is not opened before.

Every trial site is equipped first with a start package of fixed number of medication sets. The size of further packages may be adapted in accordance to the accrual of the trial sites.

The trial sites inform the ZKS about every included patient and transfer the medication ID. Matching the randomisation list with the correspondent list from the central pharmacy Erlangen, the ZKS is able to oversee the randomisation process and the medication logistics.

By study design most of the effects in secondary endpoints cannot be unequivocally attributed to the trial medication. Regarding the parameters, they are not susceptible to biased evaluation either. To minimize bias arising from evaluation of EEG-patterns, EEG evaluation trainings shall be performed. Each trial site will be equipped with working instructions to ensure homogeneity of procedures. These working instructions will cover:

1. The preparation of the intervention
2. The procedures during 60 minutes after the start of intervention
3. The procedure of uploading the EEG data
4. The report of AEs.

Blinding

Patients as well as treating physicians are blinded until 60 min after initiation of the infusion at the latest. After 60 minutes at the latest, the medication is unblinded in order to be able to decide on further treatment.

For unbiased evaluation of the primary endpoint final EEG is assessed by two expert raters (EEG reference board) blinded to treatment arm allocation.

Standardisation

Endpoints like infections are defined in detail in working instructions. Furthermore, standardisation of procedures will be achieved by providing an intervention package for each study patient containing a detailed plan with step by step guidance. Local study teams will be trained in personal sessions by members of the coordinating investigator team at initiation visits. Depending on demand, further training activity will be delivered as a webinar or telephone conference. All diagnostic EEGs are assessed retrospectively by a two blinded external expert raters 1) for quality assurance and 2) to determine protocol deviations leading to exclusion of the patient from the per-protocol analysis.

8.2 End Points

8.2.1 Primary End Point

Primary endpoint is the effectiveness of intravenous valproate (VPA) or levetiracetam (LEV) to terminate eSE and maintain control of epileptic activity **up to 60 minutes** after initiation of the trial intervention defined:

⇒ for patients with CSE: cessation of convulsions within 15 min after initiation of infusion, subsequent stable cessation of convulsions during 60 minutes after initiation of infusion.

⇒ for patients with NCSE: cessation of EEG signs of NCSE according to Salzburg criteria (Leitinger et al. 2016) within 15 min after initiation of infusion, subsequent stable cessation of ictal EEG activity according to the Salzburg criteria during 60 minutes after initiation of infusion as evaluated by the external EEG reference board.

Evaluation of this endpoint is possible for each patient.

Statistically, the time to cessation as well as the success rate is evaluated.

8.2.2 Secondary End Points

Secondary endpoints are:

Efficacy

- Time from initiation of trial intervention to cessation of eSE within 60 minutes
- Neurological status (including vigilance) 60 minutes after initiation of intervention

- Difference of blood levels of VPA and LEV before and 60 minutes after initiation of intervention
- Recurrence of seizures or nonconvulsive/ convulsive SE after initially successful intervention
- For patients who failed the primary endpoint, number of patients in whom SE cessated during 60 minutes after initiation of intervention according to the treating physician
- For NCSE patients who failed the primary endpoint, time to first cessation, as verified by EEG
- Number of patients with SE-associated ventilation until hospital discharge
- Functional outcome at discharge, defined by Barthel Index (BI) and modified Rankin Scale (mRS)

Safety

- Mortality
- Need for any emergency medication (different from allocated study drug) during 60 minutes after initiation of study intervention
- Need for ventilation (noninvasive/ invasive) during 60 minutes after initiation of intervention
- ~~Number of patients with SE-associated ventilation until hospital discharge~~
- Intrahospital complications
    - Incidence of delirium as diagnosed by the treating physician
    - Infections requiring intravenous administration of anti-infectives
    - Adverse events related to infusion/subsequent therapy with antiepileptic drug (sedation, dizziness, nausea, vomiting, thrombocytopenia, leukopenia, hypotension, new elevation of liver enzymes, hyperammonaemia,acute new liver failure or pancreatic damage, tremor, psychiatric abnormalities)

The mRS is the recommended outcome measure in stroke trials as it is directly relevant to the patient´s ability to return to daily life. Its scores range from 0 (no impairment) to 6 (death) and include special items as walking and independence that are indicative of the degree to which a patient is competent to participate in daily activities (Lees et al. 2012). As most of the seizures occurring in elder people are caused by an underlying structural brain injury the clinical phenomenology of focal convulsive SE or nonconvulsive SE may imitate stroke-related deficits. Thus, the effects on functional outcome may be quantified meaningfully and appropriately by the mRS. The Barthel Index is also a well-established clinicometric scale which is utilized to reflect elderly patient´s need for care in particular and includes basic activities such as transfer and personal hygiene (Mahoney and Barthel 1965).

The secondary endpoints will provide valuable insights into the disorder in this age group. There is a special emphasis on characteristics of SE and concomitant intrahospital complications to provide an equal evaluation of the efficacy and safety of antiepileptic treatment in the elderly.

The combination and comparison of the retrospective and prospective assessment of mRS and BI are chosen in order to quantify its consequences on functional outcome.

<u>Determination of primary and secondary measures</u>

All clinical scales (GCS, mRS, BI) are validated and widely used in clinical settings and / or clinical trials. They will be performed by trained neurologists. The pre-mRS and pre-BI reflect the functional status of the patient before the admission to the hospital and will be scored by the patient or a close relative if the patient´s capability is impaired. Recurrence of SE or seizures during the hospital stay is defined as witnessed convulsions with or without

impairment of consciousness in case of CSE and the detection of ictal EEG activity in case of NCSE. SE-associated mortality includes all cases of death during the hospital stay even if they are not directly related to SE. The necessity to invasive ventilation is defined as the proportion of patients who have to be ventilated during the hospital stay.

8.3 Statistical Description of the trial hypothesis

8.3.1 Statistical Hypotheses/Statistical Estimation Method

The trial hypotheses are:

⇒ H_0: The frequency of cessation of SE is equal in both arms.
This is equivalent to the hypothesis: Odds ratio (OR) = 1.

⇒ H_A Frequencies of cessation of SE differs between the arms.
This is equivalent to: OR ≠ 1.

Significance level is determined 5% for two-tailed tests.

8.4 Sample Size Discussion

Randomised clinical trials on treament of established Status Epilepticus are rare. The meta-analysis of Yasiry and Shorvon (2014) compares cessation of benzodiazepine resistant SE after treatment with one of five substances which included VPA and LEV. In a second meta-analysis, (Trinka et al. 2015) examined the efficacy of VPA in therapy of established SE. Third, (Alvarez et al. 2011) published results of the registry of benzodiazepine- resistant SE patients. They compared second-line treatments utilizing PHE, VPA or LEV. The reported frequencies of cessation of SE (within variable time frames) vary widely due to different study designs (RCT, prospective open label or retrospective trial, dose schemes, endpoint definition). The cessation rates in our trial with elderly patients are expected to be lower than those reported by (Yasiry and Shorvon 2014): VPA 76% and LEV 68.5%, see e.g., (Zelano and Kumlien 2012). A recent article (Misra et al. 2017) reports 70% response for VPA treatment.

New articles (Beuchat et al. 2018, Kapur et al. 2019, Sánchez Fernández et al. 2019) do not eliminate the indecision between the standard drugs for stage II. The systematic review and meta-analysis from Sánchez Fernández reports 70% success for VPA and 62% for LEV.

Omitting the studies of pediatric SE and with first-line treatment of SE, an odds ratio OR = 1.7 in favour of VPA vs. LEV is calculated, which seems much more realistic than the reported OR = 2.7 for SE cessation in (Alvarez et al. 2011). The OR = 1.7 applies for cessation frequencies of 60% (VPA) and 47% (LEV). Assuming these rates, a significance level of 5% and a power of 80%, the R (R Core Team 2017) package TrialSize calculates for chi² test a sample size N = 454.

All patients are monitored in an appropriately equipped environment (emergency room, intermediate care, intensive care unit or similar). Therefore, maximal 5% drop-outs are expected (cf. 6.4.2). Assuming a drop-out rate of about 5 %, a total of **477 patients** are to be randomized.

The secondary endpoints will provide valuable insights into the safety of both treatments. Additionally they will provide an important source for characteristics of the disorder in elderly patients. However, this analysis is mainly explorative and power estimation is not sensible.

8.5 Statistical Methods

8.5.1 Analysis Population

Primary analysis bases on the full-analysis set (FAS), that is, all patients who received trial medication. The patients are analysed in the arm they were allocated independently from the actual treatment. This follows the intention-to-treat principle.

The per-protocol set (PPS) consists of all patients who pass through the study according to the TP. Patients for whom the diagnosis NCSE was not confirmed by the central EEG review will be excluded from the PPS. Per-protocol analysis is done as a sensitivity analysis. However, if PPS is 95% or more from the FAS, a PP analysis is dispensable.

8.5.2 Planned Methods for Analysis

The primary endpoint will be analysed by means of a generalized mixed linear model with binomial link function incorporating randomisation arm as fixed and centers as random effects. Odds ratios are calculated as effect sizes incl. 95% confidence interval (CI) and tested. In secondary analysis, the cessation rates are tested by chi² test. Cessation rates and their difference with 95% CI following Wilson (Newcombe 1998b, 1998a) are calculated.

Other event rates are estimated with 95% CI (Wilson) and possibly are compared by chi-squared test without continuity correction and by mid-p test if expected numbers are too small, respectively. Time-to-cessation is analysed by time-to-event methods (Kaplan-Meier estimates and logrank test).

Latency between start of SE and 1) application of the last benzodiazepines before the start of study medication and 2) start of study medication is analysed by descriptive statistics for CSE and NCSE separately. An adjustment of the primary analysis for these covariates will be considered.

(Generalized) mixed linear models are built for the other endpoints (e.g. questionnaire scores) as well for longitudinal analyses similar to the primary endpoint.

In secondary analysis, the inclusion of the Status Epilepticus Severity Score (STESS, Rosetti et al. 2008) into multiple models for several endpoints is envisaged.

Explorative subgroup analyses are performed for female / male patients, generalized or focal CSE with impaired consciousness/ focal CSE without impaired consciousness / NCSE with coma / NCSE without coma. Although the trial is not powered for these comparisons, the analyses will give valuable new insights on the subtypes of SE and elderly patients.
Due to the short period the patient is in the study, we expect that missing data will be a minor problem. Multiple imputation is envisaged only if a high proportion of questionnaire data is missing. The definition and calculation of derived variables as well as a detailed description of the analysis method is stipulated in the statistical analysis plan. It will be established when data from 10% of the patients are input into the database. This proportion is absolutely necessary to take main properties and relationships of the data into consideration.

8.6 Statistical Monitoring

The trial conduct will be closely supervised by means of central and statistical monitoring according to ICH E6 (R2). The objectives are

- to detect safety relevant signals as soon as possible,
- to detect relevant protocol violations and to prevent their future occurrence by prompt reaction

Central and statistical monitoring bases on a thorough risk analysis. The main target is to find possible signals of danger for safety of patients and for the success of the trial. Then, actions may be taken timely to amend the trial.

Second, statistical monitoring will deliver data for the meetings of the DMC, too.

Finally, data consistence is proved by checking for extreme / unplausible values as well by cross-checking. This shall ensure a high quality of data evaluation and capturing.

Especially, these topics are important:

- patient accrual
- arm balance
- dropouts and missing values
- patient safety

8.7 Interim Analysis

No formal interim analysis for superiority or futility is planned. Even if no treatment difference can be proven, the full sample is desirable to obtain reliable estimates of effect size in the first randomized trial comparing second-line treatment with VPA and LEV in elderly patients.

8.8 Final Analysis

When all scheduled patients have finished the trial and all data are inputted the data were finally checked. This may trigger new queries that must be processed. If all queries are answered the database is closed. Then, final analysis may begin.

9 ETHICAL, LEGAL AND ADMINISTRATIVE ASPECTS

9.1 GCP-Statement

All persons participating in the conduct of the trial (sponsor, authorized representative of the sponsor, investigators, etc.) commit themselves to observe the Declaration of Helsinki of the WMA (in its current version), as well as all pertinent national laws and the ICH guidelines for Good Clinical Practice (GCP) ICH E6(R2) (EMA/CHMP/ICH/135/1995) issued in June 2017.

9.2 Initial Submission

9.2.1 Submission to the Ethics Committee and Federal Authority

Prior to submitting the trial related documents to the leading (and involved) ethics committee(s) and the responsible federal authority, the sponsor must enter the trial into the European database for clinical trials (EudraCT).

Afterwards, the protocol and all other associated documents according to GCP-V §7 will be submitted to the leading ethics committee for approval. Parallel to the submission to the leading ethics committee (EC), each participating EC is informed of the submission and also receives a copy of the documents including those of the trial sites, which they have to approve. At the same time the study documents will be submitted to the responsible federal authority (BfArM) according to the requirements of GCP-V §7.

The trial can start only after obtaining a positive review by the leading ethics committee and approval from the responsible federal authority. The written approval of the EC must be filed

in the trial master file (TMF). Additionally, every participating centre must receive a copy of these documents to be filed in the investigator site file (ISF).

9.3 Protocol Amendments

Changes made to the protocol that was appraised positively by the ethics committee and approved by the responsible federal authority must be positively reappraised and approved if the changes

- are such that they may affect the subjects' safety
- fundamental changes to the therapeutic procedures
- updates to the SmPC that may have an impact on the assessment of adverse events, including the definition of possible SUSARs
- result in further data collection that necessitates changes to the patient information and/or informed consent form,
- affect the interpretation of the scientific documents upon which the trial is based or the significance of the results of the trial,
- significantly affect the leadership or conduct of the trial, or
- concern the quality or the innocuousness of the investigational drug

Changes to the protocol may only be performed by the sponsor, the authorised representative or the coordinating investigator in co-operation with the biometrician.

After approval of the trial changes, all participating trial sites have to be informed about the changes in writing and supplied with potentially changed documents.

The whole process has to be documented in the Trial Master File (TMF).

10 DOCUMENTATION

10.1 General information and Access Rights

In the context of a database for electronic data capture only, the Case Report Form (CRF) will be designed by the ZKS Leipzig in cooperation with the Coordinating Investigator and provided as electronic form (eCRF). In order to facilitate the documentation as per protocol in case of malfunction of the electronic system or any of its components, a paper version of the CRF (interim CRF) will be provided in the ISF (investigator site file). The data on this paper version will be transferred to the eCRF as soon as the electronic system is available again.

Special CRF forms will be provided as paper CRF:

- **SAE-forms**, because these have to be sent to the ZKS Leipzig in a printable version for submission to the relevant authorities

An eCRF will be provided for each patient. The patient will be identified as per patient-ID only. The eCRF must be completed shortly after each trial visit according to ICH Guideline E6 chapter 4.9.1 and to enable central monitoring of the trial data.

Access to the data base will be limited to authorised staff only. Authorisation is granted by the site’s investigator using the trial specific staff signature and delegation log. Based on the staff signature and delegation log access to the eCRF will be granted by the responsible staff at the ZKS Leipzig.

Authorised staff members on site will be able to enter and update data as well as finalise data by electronic signature during the conduct of the trial according to a trial specific concept for documentation. This concept is based on the internal Standard Operating Procedures implemented by the ZKS Leipzig and follows the ICH Guideline E6. All entries and data changes will be tracked automatically including date, time and person who entered/changed information (audit trail). Major correction(s) or major missing data have to be explained.

10.2 Patient File and Source Data

All information required by the protocol and therefor collected during the clinical trial must be recorded by the Investigator or an authorised member of the trial team as source data in the source documentation for the trial (e.g. patient file).

Source data according to ICH Guideline E6 are defined as any information in original records and/or certified copies of original records of clinical findings, observations, or other activities in a clinical trial necessary for the reconstruction and evaluation of the trial. Source data are contained in source documents.

The Source Data Agreement is defining source data and their location for respective CRF entries. It will be filled in at the initiation visit, signed by the investigator or deputy investigator and filed in the trial master file.

In order to confirm the completeness, accuracy and consistency of the data with the data in the source documents the investigator or deputy investigator has to electronically sign each patient's CRF after his/her individual end of trial participation.

10.3 Data Management

For creation of the trial database the EDC tool secuTrial®, developed and distributed by interActive Systems GmbH (iAS), will be used. The database will be validated according to the Standard Operating Procedures (SOPs) of the ZKS Leipzig prior to data capture.

The information entered into the eCRF by the investigator or an authorised member of the trial team is systematically checked for completeness, consistency and plausibility by routines implemented in CDMS such that discrepancies can be dealt with at data entry. Errors and Warnings are listed in a validation report and can be resolved at any time during entry process. On completion of the data entry the site staff flags the eCRF-pages as 'data entry completed' (DEC).

During on-site monitoring or central/statistical monitoring, the monitor or the data manager at the ZKS Leipzig may create a manual query for discrepancies that are identified after DEC. All eCRF-pages with queries are marked in the system and a report with all queries listed is available. The site staff is responsible for data correction and can resolve queries directly in the eCRF-page.

The ZKS Leipzig will supervise and support the solution of queries and will close all correctly resolved queries. In case a query cannot be solved, the data management staff may close the query in agreement with the trial biometrician.

In case of self evident corrections by datamanagement staff, a list of self evident corrections or data entry conventions (e.g. for data entry of paper-based life questionaires) will be available and authorised by the coordinating investigator or the responsible biometrician of the clinical trial. Each trial site will receive a list of performed SECs at the end of the trial.

During the whole course of the trial, a backup of the data is made on a daily basis according to the backup policies of the ZKS Leipzig. Unauthorised access to patient data is prevented by the access concept of the trial database, which is based on a strict hirarchy and role concept.

Any change of data (e.g. when data is changed in the database during query management) is recorded automatically via audit trail within the database.

At the end of the trial, once the database has been declared complete and accurate, the database will be locked. Thereafter, any changes to the database are possible only by joint written agreement between sponsor/sponsors authorised representative/coordinating investigator, biometrician and data manager.

A pseudonymised evaluation and anonymised publication of the data is ensured.

10.4 Archiving

All relevant trial documentation (Trial Master File), the electronically stored data, the original CRFs and the final report will be stored for at least 10 years (according § 13 (10) GCP-V) by the sponsor after the trial's completion.

At the investigating sites, the investigators' files, patient identification lists, signed written consent forms, copies of all CRFs and the patients' files will be stored for at least 10 years after the trial's completion. If local rules or other legal requirements (e.g. Strahlenschutzverordnung, Röntenverordnung) require longer periods of archiving, then these are to be met.

11 REFERENCE EVALUATIONS

All study related electroencaphographical data will be evaluated by a central committee consisting of two external expert reviewers (EEG reference board) who are medical specialists in neurology and also experts for epileptology. They are blinded to the treatment; clinical data of the patient will not be provided. For the purpose of the external review every trial site transfers the pseudonymized EEG data to a study specific cloud, the related server is located at the ZKS Leipzig. Access to the EEG data is restricted to the reviewers. Each trial site is only permitted to upload data. The pseudonymisation can be realized in the following manner:

- ⇒ the clinical recording of a patient will be stopped and a new record labelled with the patient ID, will be started
- ⇒ after converting the data in an edf file the heading with the patient related information can be read out in a text editor and pseudonymisation can be checked before the transfer to the cloud

Reference evaluation of the EEGs will be completed within 1 month. Disagreement between the two expert raters will be resolved by discussion. If the EEG reference board disagrees with the diagnosis NCSE in more than 25% of all cases contributed by a given trial site over a year the trial staff of that site will undergo additional training.

12 SUPERVISION OF THE CLINICAL TRIAL

12.1 Access to Source Data

According to ICH-GCP and the applicable German laws, the investigator must permit all authorized third parties access to the trial site and the medical records of the trial subjects (source data). These include the clinical trial monitors, auditors and other authorized employees of the sponsor, as well as members of the local or federal authorities. All these persons are sworn to secrecy.

12.2 Monitoring

The ZKS Leipzig will be responsible for trial monitoring. Pre-study, initiation, regular and close-out visits will be performed in all trial sites. A risk-based monitoring strategy will be implemented, as required by ICH E6 R2 (Chapter 5.0).

During trial conduct, central and statistical monitoring procedures will be combined with on-site monitoring visits in order to achieve high protocol compliance and data quality, as well as to ensure patients' safety and rights. The choosen monitoring strategy depends on the results of the risk analysis done during the protocol development and will described in the trial specific monitoring plan.

In general, a first monitoring visit at a trial site will be scheduled after the inclusion of the site's first patient(s), checking protocol compliance and preventing further systematic errors due to misunderstandings. All trial sites will then be visited regularly. The frequency of further on-site monitoring visits will depend on the trial site's recruitment rate and on whether problems have been detected with the site, either by prior on-site visits or by central monitoring.

Prior to every scheduled on-site visit, the monitor will be provided patient synopses summarising the data already available in the database, and indicating possible protocol deviations or inconsistencies. If deemed necessary (e. g. in case of a high number of data inconsistencies/queries), queries rised by pharmacovigilance and/or from the statistical monitoring will be communicated to the site in due time before the on site visit, to enable a timely processing.

During the visits the monitor will:

- check informed consent forms of all patients enrolled
- perform source data verification of the key data (selected baseline parameters, therapy delivery, serious adverse events, follow-up) in a random sample of the site's patients
- perform targeted source data verification for patients with possible deviations
- discuss open queries raised by data management or drug safety personnel
- check essential parts of the investigator site file (see monitoring plan)
- check source data for AEs or SAEs, which have not been properly reported in the eCRF
- check for major GCP-breaches and/or protocol violations

according to the trial specific monitoring plan.

12.3 Audits

In order to guarantee that the conduct of the study is in accordance with ICH-GCP and the national laws, audits are planned to be carried out by an unbiased auditor.

By signing the protocol agreement, the investigator agrees to give the auditor access to all relevant documents for review.

The investigator should promptly notify the sponsor or its authorized representative of any audits scheduled and promptly forward copies of any audit reports received to the sponsor or its authorized representative.

12.4 Inspections

According to German Medicinal Products Act (AMG) and the corresponding GCP-guidelines (GCP-V), inspections of the trial sites may be performed by the local or federal authorities at any time during or after completion of the trial.

The investigator agrees to give the inspectors access to all relevant documents for review.

The investigator should promptly notify the sponsor or its authorized representative of any inspections scheduled by any regulatory authorities and promptly forward copies of any inspection reports received to the sponsor or its authorized representative.

12.5 Independent Supervision of the Trial

An Independent Data Monitoring Committee (DMC) will meet periodically to perform a review and an evaluation of the accumulated study data regarding:

- safety of the trial intervention
- integrity and validity of the data
- appropriate study conduct
- study progress

to guarantee the subject's safety.

The DMC will consist of three individual experts who are not involved in the ToSEE clinical trial activities and who have no conflict of interest (financial, proprietary, professional or other) with any of the participating organisations.

These core members have sufficient combined expertise in the medical disciplines at hand:

- the clinical aspects of the underlying disease (Status epilepticus)
- complications associated with the treatment of epilepsy
- biostatistics
- clinical trial conduct and methodology

Other ad hoc specialists may be invited as a non-voting member of the DMC whenever additional expertise is required.

In order to allow the DMC to fulfil its responsibilities, the ZKS Leipzig will support the DMC by providing updated data in appropriate format for analyses. In addition, the ZKS Leipzig will produce regular safety reports on the trial together with the coordinating investigator according to the regulatory requirements. These reports are also forwarded to the DMC.

While reviewing, the DMC will consider the study-specific data as well as any relevant background knowledge of treatment, the therapeutic procedures and the information provided about the patient population in the study. The DMC will specifically review:

- the quality, completeness, accuracy and timeliness of the collected data
- the collected data that may provide evidence of study-related adverse effects so far
- the performance of the individual clinical centres that are involved in the study
- the overall compliance with the study protocol and the goals for recruitment and retention
- all factors internal or external to the study that may affect the study outcome, compromise the confidentiality or the ethics of the study or impact patient safety (protocol violations, newly available scientific or therapeutic developments…)

The DMC will meet at regular intervals (once a year, preferably by telephone, but in person if needed) in open/closed sessions. The DMC will maintain the data confidentiality during all phases of the reviews and deliberations.

Following DMC meetings, the DMC will provide written recommendations to the co-ordinating investigator concerning further trial implementation (unchanged continuation, continuation with changes, interruption, termination). Such recommendations can be based on the detection of emerging negative data trends or prospects of ethical or safety guidelines not being met. The DMC may also request contacts between itself and the co-ordinating investigator by telephone or in person.

A DMC-charter will further specify the tasks of the DMC.

12.6 Data protection and Confidentiality

The sponsor, together with ZKS Leipzig and the trial sites, is responsible for the implementation and data processing in accordance with Article 4(7) of the EU Data Protection Basic Regulation 2016/679 in this trial. The ZKS Leipzig is responsible for implementation of procedures for data collection, storage, protection, retention and destruction. The ZKS Leipzig has implemented a data safety and security concept according to the requirements of the German Federal Office for Information Security (www.bsi.bund.de).

All data will be initially collected by investigators in the recruiting trial sites. Together with information on the trial, eligible patients will be informed about data capture, transmission, analysis processes and their rights according to the General Data Protection Regulation (GDPR). Once a patient is eligible and has given his/her informed consent to trial participation and data collection, the investigator will assign the patient a unique patient identification code. Patient identification code lists will be generated in advance by ZKS Leipzig and forwarded to the recruiting sites. These lists are part of the investigator site file and remain at the recruiting site. These lists are the only documents that allow for re-identification of the patients.

All clinical data entered by the investigators (or their designated staff) into eCRFs will be recorded in a pseudonymized form (i.e. without reference to the patient’s name and date of birth) exclusively using the patient’s identification code.

Clinical monitors appointed by ZKS Leipzig will regularly visit the recruiting sites and verify the informed consent forms. Data will only be used for analysis after it has been verified by monitors that the patient has unambiguously given his or her consent for trial participation as well as for data capture, transmission and analysis. The patients are informed of this fact and agree to the procedure with the patient information/informed consent.

In the event of withdrawal of consent, the necessity for storing data will be evaluated. While the General Data Protection Regulation (EU) 2016/679 strengthens personal data protection rights, encompassing the right to access, rectification and withdrawal of data, it also specifies the situations when restriction on those rights may be imposed. The withdrawal of informed consent should not affect the results of activities already carried out, such as the storage and use of data obtained on the basis of informed consent before.

In the ToSEE trial patients are unable to give an informed consent at the timepoint of the inclusion. For this reason, patients are included in the clinical trial as part of emergency treatment, after confirmation by an independent medical consultant or with the consent of a legal representative or authorised representative. After inclusion and treatment of the patient in the trial, the following scenarios are possible:

1) After reaching the ability to consent to the trial, the patient refuses further trial participation.

2) Legal or authorized representative refuse further trial participation. This is the case if a legal care relationship is established subsequently **or** if it becomes known after the start of trial treatment that a legal or authorized care relationship already exists and the legal/authorised representative was informed later.

3) The patient dies without give an informed consent.

In the aforementioned cases, the necessity of the data already collected will be evaluated

according to the regulatory requirements of §40 (3) AMG. Stored data may continue to be used, where necessary, in order to:

- to determine the effects of the medicinal product to be tested,
- to ensure that the patient's legitimate interests are not compromised,
- comply with the obligation to submit complete registration documents.

12.7 Declaration regarding Data Protection

We hereby confirm that all clinical trial information will be recorded, processed, handled and stored by ZKS Leipzig, Härtelstr. 16-18, 04107 Leipzig, Germany on behalf of the sponsor.

Data captured by the investigators will be processed in such a way that it can be accurately reported, interpreted and verified while the confidentiality of records and the personal data of the subjects remain protected. Data capture and processing will be in accordance with the applicable law on personal data protection and with the “General Data Protection Regulation” (EC) 2016/679 of the European parliament and of the council.

Access to the data is strictly limited to authorised persons. Data are protected against unauthorised access.

12.8 Declaration regarding the Pseudonymized Transfer of Personal Data

The sponsor certifies herewith that the transfer of pseudonymized personal data will take place according to the documentation and communication regulations in §§ 12 und 13 of the GCP-guidelines (GCP-V). Moreover, the sponsor certifies that trial participants who do not permit the transfer of data will not be admitted to the trial. This will be ascterained by including the relevant information in the patient information/informed consent.

12.9 Anonymisation of Data after the end of Archiving

After the end of the archiving period, all clinical data present at the ZKS Leipzig will be stored in an anonymous form.

All data will be subject to an anonymization process removing personalised data as far as possible, i.e. without endangering the possibility to answer scientific questions related to the trial. Anonymised data will be relocated to a separate, access restricted, file location and secondary data sources will be deleted.

Non clinical data (like contact information) will be deleted after the end of the archiving period.

13 ADMINISTRATIVE AGREEMENTS

13.1 Adherence to the Protocol

The clinical trial described here will be conducted and analyzed in accordance with local laws (AMG / GCP-Verordnung) and ICH guidelines for Good Clinical Practice (GCP).

Protocol violations are all deviations from the protocol defined procedures as outlined in the risk analysis and resulting in a list of major protocol violations which will be analysed if occurred in study patients and considered in final data analyses (see sec 8.5.1).

After a patient has been enrolled, it is the investigator's responsibility to avoid protocol violations in order to obtain unbiased data for the trial.

Those protocol violations deemed to be major are defined by the risk analysis performed before and during trial implementation and will be further detailed in separate documents belonging to the risk assessment/monitoring plan. This list can be augmented in the course of the trial. Major protocol violations will be reported to the ZKS Leipzig, which will inform the sponsor.

All protocol violations will be documented and discussed with the responsible biometrician before closing the data base and carrying out the statistical analysis.

The investigator must ensure that the recorded data are documented as per protocol. Minor variations are inevitable in clinical routine, but must be documented together with a justification.

13.2 Funding and Insurance

The trial is funded by:
Bundesministerium für Bildung und Forschung (BMBF), Förderkennzeichen 01GL1804

Patients are insured at the insurance company HDI-Gerling Industrie Versicherung AG, Niederlassung Leipzig; Eisenbahnstr. 1-3; 04315 Leipzig. The number of the insurance policy is: 28 - 138971 03302. The maximum insurance sum for the individual insured person is 500.000 €. The maximum insurance sum for all insured events of the clinical trial is 50.000.000 €.

A copy of the insurance policy and general insurance conditions (AVB) can be obtained from the investigator site file.

13.3 Notification of the Local Authorities

Prior to enrolment of the first patient in the trial, the sponsor, his/her legal representatives/ contractors and all investigators and their deputies are responsible according to German Medicinal Products Act AMG §67 (1) and the requirements of the GCP-V §12 and 13 for notifying the local regulatory authority of their participation in the trial.

According to §67 (3) AMG and §§ 12,13 GCP-V the sponsor, his/her legal representatives/ contractors and all investigators and their deputies are also responsible for notifying the local regulatory authority of amendments, premature termination of trial arms or of the whole study and the regular trial termination.

13.4 Publication Policy and Registration

The results of this trial will be submitted for publication in a peer-reviewed, international English-language journal of appropriate aim and scope. Accordingly, the clinical trial will be registered at DRKS before recruitment starts. According to the results of main project, the results will be submitted in separate or combined manuscripts; decisions about the form and scope of individual manuscripts will be discussed among all persons participating in the design, conduct and analysis of the study who qualify for authorship. The coordinating investigator together with the biometrician is responsible for drafting and circulating manuscripts and for discussing and handling requests by co-authors or/and sponsors to edit the text.

The authorship will follow the criteria for authorship developed by the International Committee of Medical Journal Editors (ICMJE), including those that distinguish authors from other contributors.

The ICMJE recommends that authorship be based on the following 4 criteria:

- Substantial contributions to the conception or design of the work; or the acquisition, analysis, or interpretation of data for the work; AND
- Drafting the work or revising it critically for important intellectual content; AND
- Final approval of the version to be published; AND
- Agreement to be accountable for all aspects of the work in ensuring that questions related to the accuracy or integrity of any part of the work are appropriately investigated and resolved.

All those designated as authors should meet all four criteria for authorship, and all who meet the four criteria should be identified as authors. Those who do not meet all four criteria will be acknowledged in the manuscript.

The scientific use of data resulting from this trial bay local trial sites is ruled by the site contracts between the sponsor and the local trial sites. Generally, sites might use data for own scientific questions (independent from the questions discussed in this trial protocol) and publication after consultation with the sponsor.

13.5 Data Sharing Statement

According to the recommendations on data sharing by the International Committee of Medical Journal Editors (ICMJE) data resulting from the ToSEE-trial will be made available to the scientific community as follows:

After publication of the major results and upon reasonable request from researchers performing an individual patient data meta-analysis, individual patient data that underlie published results will be shared after de-identification. This requires approval by the local Institutional Review Board (IRB) of the researcher requesting the data along with public registration of the meta-analysis.

Summary statistics that go beyond the scope of published material will be made available to researchers for meta-analysis upon reasonable request and if the necessary data analysis is not unduly time-consuming. Together with publication of the main results, the trial protocol in full will be made publically available as well as the statistical analysis plan.

14 PROTOCOL SIGNATURES

Confirmation of the Final Protocol

We hereby certify that this is the final version of the protocol:

Authorized representative of the sponsor:	18.5.2020	[illegible]
	Date	Signature
Biometrician:	18.05.2020	[illegible]
	Date	Signature

15 PROTOCOL AGREEMENT

Herewith I declare that I have read and understood the present protocol and agree to honour each part of it. I will ensure that all the patients enrolled in the trial by my site will be treated, observed and documented in accordance with this protocol. I will ensure that all persons assisting with the study under my supervision are adequately informed about the protocol, the investigational product and their duties.

Centre-ID	ToSEE - __/__/
Address trial site (stamp)	

Date

Signature Investigator

Date

Signature Deputy Investigator (***Germany only***, *acc. to national law)*

16 APPENDIX

16.1 Classification of Adverse Events

16.1.1 Degree of Seriousness

The seriousness of an Adverse Event will be determined in accordance with the definitions in 7.1 and 7.4.

16.1.2 Assessment of Intensity/Severity

The assessment of the intensity accords with CTCAE V5.0

Mild Adverse Event	• asymptomatic or mild symptoms;<br>• clinical or diagnostic observations only;<br>• intervention not indicated.
Moderate Adverse Event	• minimal, local or noninvasive intervention indicated;<br>• limiting age-appropriate instrumental ADL*[3].
Severe Adverse Event	• medically significant but not immediately life-threatening;<br>• hospitalisation or prolongation of hospitalisation indicated;<br>• disabling;<br>• limiting self care ADL**
Life-threatening Adverse Event	• Life-threatening consequences;<br>• urgent intervention indicated
Death related to Adverse Event	

16.1.3 Determining the Causal Relationship

The investigator/ the deputy or the authorised medical staff must assess whether or not the Adverse Event is causally related to the administration of the trial medication. The following classification is to be used.

- Reasonable possibility
- No reasonable possibility

A reasonable possibility exists, if one of the following WHO-UMC criteria is met:

[3] **Activities of Daily Living (ADL):**
*Instrumental ADL refer to preparing meals, shopping for groceries or clothes, using the telephone, managing money, etc.
**Self care ADL refer to bathing, dressing and undressing, feeding self, using the toilet, taking medications, and not bedridden.

- occurring in a plausible time relationship to drug administration, and which cannot be explained by concurrent disease or other drugs or chemicals. The response to withdrawal of the drug (dechallenge) should be clinically plausible. The event must be definitive pharmacologically or phenomenologically, using a satisfactory rechallenge procedure if necessary.
- with a reasonable time sequence to administration of the drug, unlikely to be attributed to concurrent disease or other drugs or chemicals, and which follows a clinically reasonable response on withdrawal (dechallenge). Rechallenge information is not required to fulfil this definition.
- with a reasonable time sequence to administration of the drug, but which could also be explained by concurrent disease or other drugs or chemicals. Information on drug withdrawal may be lacking or unclear.
- more data is essential for a proper assessment or the additional data are under examination
- cannot be judged because information is insufficient or contradictory, and which cannot be supplemented or verified

No reasonable possibility exists, if the following WHO-UMC criterion is met:

- with a temporal relationship to drug administration which makes a causal relationship improbable, and in which other drugs, chemicals or underlying disease provide plausible explanations.

16.1.4 Expected/Unexpected

Adverse Events are unexpected if they do not occur in the manner or with the intensity described in the SmPC/Investigator's Brochure (see investigator's files).

16.1.5 Outcome of an Adverse Event

The outcome of an Adverse Event is classified as follows:

- recovered/resolved
- recovering/resolving
- not recovered/not resolved
- recovered/resolved with sequel
- fatal*
- unknown

*Note: A patient's death is not in itself an event, but the consequence of one. The event that led to the patient's death must be documented completely and reported even if death occurs four weeks after stopping medication and independent of whether or not there is a relation to the therapy or not.

16.2 Acronyms

AE	Adverse Event
AMG	Arzneimittelgesetz
BfArM	Bundesinstitut für Arzneimittel und Medizinprodukte
BI	Barthel Index
BOB	Bundesoberbehörde
CI	Confidence interval
CRF	Case report form
CSE	Convulsive status epilepticus
EC	Ethics committee
FAS	Full Analysis set
GCP	Good Clinical Practice
GCP-V	GCP-Verordnung
GCS	Glasgow Coma Scale
ICH	International Conference on Harmonisation
i.v.	intravenous
LEV	Levetiracetam
MPG	Medizinproduktegesetz
mRS	modified Ranking Scale
NCSE	Non-convulsive status epilepticus
NIHSS	National Institutes of Health Stroke Scale
PPS	Per-protocol set
RCT	Randomized Controlled Trial
SAE	Serious adverse event
SAR	Serious adverse reaction
SE	Status epilepticus
SUSAR	Suspected unexpected serious adverse reaction
TMF	Trial master file
TP	Trial protocol
VPA	Valproate
WHO-UMC	World Health Organization – Uppsala Monitoring Centre
ZKS Leipzig	Zentrum für Klinische Studien Leipzig

16.3 Template trial protocol

This trial protocol was written based on a template by the ZKS Leipzig based on the SOPs of the ZKS Leipzig.

The used template version is: Final 9.0 from 20-01-2020.

17 REFERENCES

[1] Alldredge BK, Gelb AM, Isaacs SM, Corry MD, Allen F, Ulrich S, et al. A comparison of lorazepam, diazepam, and placebo for the treatment of out-of-hospital status epilepticus. N Engl J Med. 2001 Aug 30;345(9):631-7

[2] Alvarez V, Januel JM, Burnand B, Rossetti AO. Second-line status epilepticus treatment: comparison of phenytoin, valproate, and levetiracetam. Epilepsia. 2011 Jul;52(7):1292-6

[3] Beuchat I, Novy J, Rossetti AO. Newer Antiepileptic Drugs for Status Epilepticus in Adults: What's the Evidence? CNS Drugs. 2018 Mar;32(3):259-267

[4] Brigo F, Nardone R, Tezzon F, Trinka E. Nonintravenous midazolam versus intravenous or rectal diazepam for the treatment of early status epilepticus: a systematic review with meta-analysis. Epilepsy Behav: E&B 2015 August 49;325–36

[5] Brigo F, Del Giovane C, Nardone R, Trinka E, Lattanzi S. Intravenous antiepileptic drugs in adults with benzodiazepine-resistant convulsive status epilepticus: A systematic review and network meta-analysis. Epilepsy Behav. 2019 Dec;101 (Pt B):106466

[6] Cancer Therapy Evaluation Program, Common Terminology Criteria for Adverse Events, Version 3.0, DCTD, NCI, NIH, DHHS; March 31, 2003 (http://ctep.cancer.gov), Publish Date: December 12, 2003

[7] Chu SS, Wang HJ, Zhu LN, Xu D,Wang XP, Liu L. Therapeutic effects of intravenous levetiracetam in status epilepticus: A meta-analysis and systematic review. Seizure 2020 Jan; 74:49-55.

[8] Contin M, Mohamed S, Albani F, Riva R, Baruzzi A. Levetiracetam clinical pharmacokinetics in elderly and very elderly patients with epilepsy. Epilepsy Res. 2012 Feb;98(2-3):130-4

[9] DeAssis TM, Costa G, Bacellar A, Orsini M, Nascimento OJ. Status epilepticus in the elderly: epidemiology, clinical aspects and treatment. Neurol Int. 2012 Oct 5;4(3)

[10] Declaration of Helsinki: Guiding Physicians in Biomedical Research Involving Human Subjects. Adopted by the 18th World Medical Assembly, Helsinki (Finland), June 1964. Last amendment by the 48th General Assembly, Somerset West (Rep. of South Africa) 1996

[11] DeLorenzo RJ, Towne AR, Pellock JM, Ko D. Status epilepticus in children, adults, and the elderly. Epilepsia. 1992; 33 (Suppl. S4):S15–S25.

[12] Devinsky O, Leppik I, Willmore LJ, Pellock JM, Dean C, Gates J, et al. Safety of intravenous valproate. Ann Neurol. 1995;38:670-4

[13] DeWolfe JL, Knowlton RC, Beasley MT, Cofield S, Faught E, Limdi NA. Hyperammonemia following intravenous valproate loading. Epilepsy Res. 2009;85:65–71

[14] European Commission (2004.04): Detailed guidance on the collection, verification and presentation of adverse reaction reports arising from clinical trials on medicinal products for human use, revision 2

[15] Fachinformation Levetiracetam UCB® 100mg/ml Konzentrat zur Herstellung einer Infusionslösung. Available from: URL: https://www.fachinfo.de/suche/fi/014986

[16] Farrokh S, Bon J, Erdman M, Tesoro E. Use of Newer Anticonvulsants for th Treatment of Status Epilepticus. Pharmacotherapy. 2019 Mar;39(3):297-316

[17] Foley KT, Bugg KS. Separate episodes of delirium associated with levetiracetam and amiodarone treatment in an elderly woman. Am J Geriatr Pharmacother. 2010 Apr;8(2):170-4

[18] Gebrauchsinformation Orfiril® 100mg/ml Injektionslösung. Available from: URL:https://www.desitin.de/fileadmin/user_upload/de/Produkte/PDF/GIs/Epilepsie/Orfiril_i.v_GI.pdf

[19] Gesetz über den Verkehr mit Arzneimitteln (Arzneimittelgesetz - AMG) zuletzt geändert durch das Gesetz zur Änderung arzneimittelrechtlicher und anderer Vorschriften vom 19.10.2012 BGBl. I S. 2192 (Nr. 50); Geltung ab 26.10.2012

[20] Gilad R, Izkovitz N, Dabby R, Rapoport A, Sadeh M, Weller B, Lampl Y. Treatment of status epilepticus and acute repetitive seizures with i.v. valproic acid vs phenytoin. Acta Neurol Scand. 2008 Nov;118(5):296-300

[21] Glauser T, Shinnar S, Gloss D, Alldredge B, Arya R, Bainbridge J, et al. Evidence-Based Guideline: Treatment of Convulsive Status Epilepticus in Children and Adults: Report of the Guideline Committee of the American Epilepsy Society. Epilepsy Curr. 2016 Jan-Feb;16(1):48-61

[22] Hirsch LJ, LaRoche SM, Gaspard N, Gerard E, Svoronos A, Herman ST, et al. American Clinical Neurophysiology Society´s Standardized Critical Care EEG Terminology: 2012 version. J Clin Neurophysiol. 2013 Feb;30(1):1-27

[23] Hwang ES, Siemianowski LA, Sen S, Patel R. Levetiracetam: an unusual cause of delirium. Am J Ther. 2014 Nov-Dec;21(6):e225-8

[24] International Conference on Harmonisation of Technical Requirements for the Registration of Pharmaceutical Products for Human Use: ICH Harmonized Tripartite Guideline, “Guideline for Good Clinical Practice”. Recommended for Adoption at Step 4 of the ICH Process on 1 May 1996. www.ifpma.org/ich5e.html#GCP

[25] International Conference on Harmonisation of Technical Requirements for the Registration of Pharmaceutical Products for Human Use: ICH Harmonized Tripartite Guideline, “Clinical Data Safety Management: Definitions and Standards for Expedited Reporting”. Recommended for Adoption at Step 4 of the ICH Process on 27 May 1994. www.ifpma.org/ich5e.html#Safety

[26] Kapur J, Elm J, Chamberlain JM, Barsan W, Cloyd J, Lowenstein D, et al. Randomized Trial of Three Anticonvulsant Medications for Status Epilepticus. N Engl J Med. 2019 Nov 28;281(22):2103-2113

[27] Kellinghaus C, Rossetti AO, Trinka E, Lang N, May TW, Unterberger I, et al. Factors predicting cessation of status epilepticus in clinical practice: Data from a prospective observational registry (SENSE). Ann Neurol. 2019 Mar;85(3):421-432

[28] Knake S, Rosenow F, Vescovi M, Oertel WH, Mueller HH, Wirbatz A, et al. Incidence of status epilepticus in adults in Germany: a prospective, population-based study. Epilepsia. 2001 Jun;42(6):714-8

[29] Kumar N, Swaroop HS, Chakraborty A, Chandran S. Levetiracetam induced acute reversible psychosis in a patient with uncontrolled seizures. Indian J Pharmacol. 2014 Sep-Oct;46(5):560-1

[30] Lees KR, Bath PM, Schellinger PD, Kerr DM, Fulton R, Hacke W, et al. European Stroke Organization Outcomes Working Group. Contemporary outcome measures in acute stroke research: choice of primary outcome measure. Stroke. 2012 Apr;43(4):1163-70

[31] Legriel S, Brophy GM. Managing Status Epilepticus in the Older Adult. J Clin Med. 2016 May 11;5(5)

[32] Leitinger M, Trinka E, Gardella E, Rohracher A, Kalss G, Qerama E, et al. Diagnostic accuracy of the Salzburg EEG criteria for non-convulsive status epilepticus: a retrospective study. Lancet Neurol. 2016 Sep;15(10):1054-62

[33] Leitlinien, Deutsche Gesellschaft für Neurologie. Available from: URL: http://www.dgn.org; AWMF-RNr: 030/079

[34] Madzar D, Geyer A, Knappe RU, Gollwitzer S, Kuramatsu JB, Gerner ST, et al. Association of seizure duration and outcome in refractory status epilepticus. J Neurol 2016;263(March (3))485–91

[35] Mahoney FI, Barthel DW. Functional Evaluation: The Barthel Index. Md State Med J. 1965 Feb;14:61-5

[36] Minicucci F, Ferlisi M, Brigo F, Mecarelli O, Meletti S, Aguglia U, et al. Management of status epilepticus in adults. Position paper of the Italian League against Epilepsy. Epilepsy Behav. 2020 Jan;102:106675

[37] Misra UK, Dubey D, Kalita J. A randomized controlled trial of lacosamide versus sodium valproate in status epilepticus. Epilepsia. 2017 Feb 18.

[38] Perrenoud M, André P, Buclin T, Decosterd LA, Rossetti AO, Novy J. Levetiracetam circulating concentrations and response in status epilepticus. Epilepsy Behav. 2018 Nov;88:61-65

[39] Ramsay RE, Cantrell D, Collins SD, Walch JK, Naritoku DK, Cloyd JC, et al. Safety and tolerance of rapidly infused Depacon. A randomized trial in subjects with epilepsy. Epilepsy Res. 2003;52:189–201.

[40] Rossetti AO, Logroscino G, Milligan TA, Michaelides C, Ruffieux C, Bromfield EB. Status Epilepticus Severity Score (STESS): a tool to orient early treatment strategy. J Neurol. 2008 Oct;255(10):1561-6

[41] Silbergleit R, Durkalski V, Lowenstein D, Conwit R, Pancioli A, Palesch Y, et al. Intramuscular versus intravenous therapy for prehospital status epilepticus. N Engl J Med. 2012 Feb 16;366(7):591-600

[42] Sinha S, Naritoku DK. Intravenous valproate is well tolerated in unstable patients with status epilepticus. Neurology. 2000 Sep 12;55(5):722-4.

[43] Strzelczyk A, Zöllner JP, Willems LM, Jost J, Paule E, Schubert-Bast S, et al. Lacosamide in status epilepticus: Systematic review of current evidence. Epilepsia. 2017 Jun;58(6):933-950

[44] Sutter R, Marsch S, Fuhr P, Kaplan PW, Ruegg S. Anesthetic drugs in status epilepticus: risk or rescue? A 6-year cohort study. Neurology. 2014;82:656–64.

[45] Treiman DM, Meyers PD, Walton NY, Collins JF, Colling C, Rowan AJ et al. A comparison of four treatments for generalized convulsive status epilepticus. Veterans Affairs Status Epilepticus Cooperative Study Group. New England Journal of Medicine. 1998 Sep 17;339(12):792-8

[46] Trinka E, Kälviäinen R. 25 years of advances in the definition, classification and treatment of status epilepticus. Seizure. 2017 Jan;44:65-73

[47] Trinka E, Höfler J, Leitinger M, Brigo F. Pharmacotherapy for Status Epilepticus. Drugs. 2015 Sep;75(13):1499-521

[48] Trinka E, Höfler J, Zerbs A, Brigo F. Efficacy and safety of intravenous valproate for status epilepticus: a systematic review. CNS Drugs. 2014 Jul;28(7):623-39

[49] Verordnung über die Anwendung der Guten Klinischen Praxis bei der Durchführung von klinischen Prüfungen mit Arzneimitteln zur Anwendung am Menschen (GCP-Verordnung - GCP-V) vom 19.10.2012 BGBl. I S. 2192 (Nr. 50); Geltung ab 26.10.2012

[50] Yasiry Z, Shorvon SD. The relative effectiveness of five antiepileptic drugs in treatment of benzodiazepine-resistant convulsive status epilepticus: a meta-analysis of published studies. Seizure. 2014 Mar;23(3):167-74

[51] Zelano J, Kumlien E. Levetiracetam as alternative stage two antiepileptic drug in status epilepticus: a systematic review. Seizure. 2012 May;21(4):233-6

[52] Sánchez Fernández et al. Meta-analysis and cost-effectiveness of second-line antiepileptic drugs for status epilepticus. Neurology 2019;92:e2339-e2348
